# Biodegradable PLA/PHB Composites with Inorganic Fillers and Modifiers

**DOI:** 10.3390/polym17202721

**Published:** 2025-10-10

**Authors:** Jozef Feranc, Martina Repiská, Roderik Plavec, Katarína Tomanová, Michal Ďurfina, Zuzana Vanovčanová, Ida Vašková, Leona Omaníková, Mária Fogašová, Slávka Hlaváčiková, Ján Kruželák, Zuzana Kramárová, Eduard Oswald, Pavol Alexy

**Affiliations:** 1 Institute of Natural and Synthetic Polymers, Faculty of Chemical and Food Technology, Slovak University of Technology in Bratislava, Radlinského 9, 812 37 Bratislava, Slovakia; roderik.plavec@stuba.sk (R.P.); katarina.tomanova@stuba.sk (K.T.); michal.durfina@stuba.sk (M.Ď.); zuzana.vanovcanova@stuba.sk (Z.V.); ida.vaskova@stuba.sk (I.V.); leona.omanikova@stuba.sk (L.O.); maria.fogasova@stuba.sk (M.F.); slavka.hlavacikova@stuba.sk (S.H.); jan.kruzelak@stuba.sk (J.K.); zuzana.kramarova@stuba.sk (Z.K.); eduard.oswald@stuba.sk (E.O.); pavol.alexy@stuba.sk (P.A.); 2 Audia Plastics s.r.o., Voderady 426, 919 42 Voderady, Slovakia; martina.repiska@audia.com

**Keywords:** biodegradable polymer blend, polyhydroxybutyrate, polylactic acid, inorganic filler, modifier, degradation

## Abstract

The work is focused on the study of the influence of different types of inorganic fillers, in combination with modifiers, on the rheological, thermal, and mechanical properties of a biodegradable mixture based on PLA/PHB. Ten types of inorganic fillers based on talc, magnesium hydroxide, aluminum hydroxide, calcium carbonate, and silicon dioxide were used in the study, along with three types of modifiers. It was concluded that fillers containing reactive OH groups on their surface act as strong pro-degradants in PLA/PHB blends, and their degrading effect can be suppressed by the addition of reactive modifiers. Each modifier acts specifically with different types of fillers. Therefore, it is necessary to select a suitable filler/modifier combination not only for fillers with different chemical compositions but also for fillers with different morphologies within the same chemical type. Moreover, the preparation of PLA/PHB/magnesium hydroxide blends with suitable processing and application properties opens the possibility of developing environmentally friendly polymeric materials with a reduced flammability. The addition of talc, which has a platelet structure, can increase the barrier properties of the mixture.

## 1. Introduction

In recent years, there has been an increase in interest in biodegradable polymers due to the inevitable and impending depletion of fossil resources, the processing of which places a burden on the environment, and due to technologically and financially demanding recycling methods [1,2]. Biodegradable polymers derived from renewable raw materials represent a new generation of materials that reduce the impact on the environment (energy consumption, emissions and greenhouse gases, less dependence on fossil fuels, and others). Their development is currently at its peak, but for successful applications on the market there are some limitations in use and processing (high price). Many polymeric materials have been developed based on renewable resources [3].

In the field of biopolymers, polylactic acid (PLA) and polyhydroxybutyrate (PHB) and its blends are extensively studied biomaterials. PLA is a linear, thermoplastic, aliphatic biopolyester with many advantageous properties such as biodegradability, biocompatibility, high transparency, strength, relatively low flammability, easy processing, and commercial availability, making it one of the most widely used biodegradable polyesters. Despite these advantages, PLA also has some disadvantages, such as brittleness, high gas and vapor permeability, low melt strength, and limited thermal stability [3,4,5,6]. Moreover, its biodegradation rate is slower compared to other common natural organic wastes, such as food or garden residues, which significantly limits its adoption in industrial food and yard composting facilities and its widespread use in general [7]. PHB is a biodegradable, biocompatible, thermoplastic, semicrystalline linear polyester, which is formed as an intracellular product of microorganisms (Bacillus safensis, Bacillus megaterium, and others) in the form of granules [8,9]. PHB is relatively resistant to abiotic hydrolytic degradation and is insoluble in water. The physical properties of polyhydroxybutyrate are related to its stereochemistry. The temperature characteristics of polyhydroxybutyrate include a high melting point (173–180 °C), a glass transition temperature of about 1–5 °C, and a thermal degradation temperature of about 200 °C, which is close to the melting point and causes a processing problem [9,10,11,12,13].

Blending PLA with PHB has been widely explored as a modification of the brittleness of PLA with the stiffness of PHB. However, although these two polyesters are only partially miscible, they can form blends with improved performances when appropriate compatibilization strategies are applied [14,15,16]. The PLA/PHB mixture (75/25 weight percent (wt%)) has better mechanical and barrier properties than pure polylactic acid. As the amount of polyhydroxybutyrate in this mixture increases, there is a decrease in the mechanical properties (tensile strength, Young’s modulus), but also a decrease in the glass transition temperature. The crystallization of the polyhydroxybutyrate in the mixture produces small spherulites which can act as nucleating agents for polylactic acid, leading to an increase in crystallinity [10,11]. Polylactic acid is more stable against enzymatic hydrolysis than polyhydroxybutyrate, so during biodegradation polyhydroxybutyrate primarily degrades in this mixture. In the manufacture of films from such a blend, the addition of plasticizers is necessary to reduce their brittleness, improve ductility and processability, and facilitate surface hydrolysis during biodegradation [10]. Biodegradable polylactic acid and polyhydroxybutyrate can be mixed with materials from natural sources such as starch, cellulose, cellulose derivatives, or chitin. Such preparation of the polymer mixture will ensure a reduction in the financial costs associated with their production and is an excellent alternative to the development of new materials with better properties [10,11]. The existence of weak van der Waals interactions between macromolecules in the polymer is often the reason for their immiscibility, so their compatibilization is necessary [17]. Compatibility is the specific way in which the stability of a polymer blend is achieved. The most important task is to reduce the size of the dispersed component (phase) by reducing the interphase voltage and preventing the coalescence of the dispersed particles, thereby stabilizing the system [18]. The compatibility can be improved by the interactions between dispersed components and the matrix resulting from the use of compatibilizers, which are mostly macromolecular substances with interphase activity in heterogeneous mixtures [10].

The utilization of natural origin inorganic fillers is possible as well. Calcium carbonate is an inorganic filler example that is widely used in the filling of biodegradable polymer composites, with the aim of increasing their strength, stiffness, and durability. It also reduces the overall costs of composite production and improves biodegradability, which is why its use to produce disposable products and packaging is so developed. Other investigated inorganic fillers in connection with PLA include metal oxides such as zinc oxide and titanium dioxide. These, in addition to improving strength and hardness, can add antibacterial properties. Because of this property, this type of PLA composite is being investigated for biomedical applications. Also, the addition of TiO_2_ to PHB increases its resistance to ultraviolet (UV) radiation, which is important for outdoor applications and packaging. The addition of this filler to PLA promotes crystallization, thereby improving the thermal stability of the matrix. An increase in the glass transition temperature and a slight increase in melting temperature supports the thermal resistance of the PLA composite [19,20,21]. The dimensional thermal stability of PLA can also be improved by the addition of TiO_2_ nanoparticles, which influence its crystallization. However, mechanical tests reveal that this filler contributes to a decrease in the tensile strength and an increased brittleness of the PLA material. Results also indicate that the addition of TiO_2_ leads to more intense photodegradation compared to pure PLA, which may be a useful property for regulating biodegradation [22]. Magnesium oxide (MgO) has similar effects on the PLA matrix as the previously mentioned oxides. Up to 2 wt%, there is a gradual increase in the Young’s modulus and tensile strength of the PLA composite. An adequate surface interaction between PLA and the filler ensures a stress transfer from the matrix to the filler, leading to improved mechanical properties [23]. The magnesium oxide also affects the processability of PLA through extrusion and injection molding. In addition, it reduces the brittleness, increases the thermal stability, and improves the mechanical strength and stiffness of PLA and PHB and has antibacterial properties [19,20]. The addition of silicon dioxide (SiO_2_) in polymer blends presents suitable properties, such as abrasion, optical ultraviolate (UV) filtering, luminescence, and biocompatibility, which have never been observed in their bulk state. It has a low refractive index and a high degree of thermal and mechanical stability [24]. Prapruddivongs et al. [25] studied the biodegradation behavior of PLA. They crosslinked PLA filled with precipitated SiO_2_ (commercial SiO_2_) and SiO_2_ from rice husk ash and found that SiO_2_ incorporation has a direct effect on the composites’ stability. According to Opaprakasit et al. [26], PLA/silica composites can improve gas permeability and selectivity. Materials had a strong potential to be used as biodegradable packaging films with a tunable gas permeability. The study of [24] aimed to improve the understanding of how PLA and nano-SiO_2_ interact, resulting in the specific morphology and surface properties of the blends. It was found that the mechanical properties of the blends were improved by the addition of nanoparticles. In addition, the results showed that nanoparticles have a positive influence on thermal stability as well as the thermal degradation of the blends obtained.

Among many others, talc is a filler that improves the thermal stability, mechanical properties, and processability of PLA and PHB. It reduces their brittleness, which is very important for packaging materials and industrial products. It was observed that talc acted as a nucleating agent and increased the crystallinity in polymers, and, thereby, the oxygen permeability (OTR) and the water vapor transmission rate (WVTR) were similarly reduced by talc [27,28]. The study of [29] found that the addition of talc increased the miscibility of PLA and the polyester while acting as a nucleating agent that improved PLA crystallinity. Jain et al. [30] reported that the oxygen and water vapor barrier properties of composite films were improved by 33 and 25%, respectively, at a 3 wt% talc loading. When talc was only emended in PLA, the tensile strength and modulus increased with the amount of talc, whereas Young’s modulus decreased. It was also observed that talc improved the oxygen resistance by 72% in poly(3-hydroxybutyrate-co-3-hydroxyvalerate) (PHBV), and by 62% in PLA matrices. Compared to PLA itself, PLA/talc films also displayed a better barrier to water vapor (up to 55% improvement). Similarly, oxygen permeability is also reduced with talc addition [30,31]. With the blending of 20 wt% PHBV, an overall further improvement of about 80% in both properties was achieved. In addition, it has been reported that mixing 20–35 wt% of PHBV with PLA is a suitable combination to achieve high barrier properties to both oxygen and water vapor, while maintaining the biocompatibility of the material [32].

In addition to improving mechanical properties, fillers can also impart a certain level of flame-retardant properties. These fillers include organically modified montmorillonite (OMMT), metal hydroxides such as magnesium hydroxide (Mg(OH)_2_) and aluminum hydroxide (Al(OH)_3_), phosphorus derivatives, etc. However, while these fillers can enhance the flame-retardant characteristics of PLA, they may also induce negative effects, such as a loss of mechanical and thermal properties and degradation of the PLA matrix [33,34,35,36]. The metal hydroxides have advantages such as non-toxicity, reducing the acidity of the combustion products and reducing the temperature of the polymer due to their ability to act as a smoke suppressant. However, they need to be added in large amounts (more than 30 wt% to 60 wt%) to make them effective as flame-retardant agents, which may affect the deterioration of the mechanical properties of the final products [37,38].

To avoid this negative phenomenon, the utilization of reactive modifiers in blends was our focus. Reactive modifiers are widely used in biodegradable polyesters to improve melt stability, rheology, and toughness by counteracting chain scission, inducing branching, and enhancing interfacial compatibility. They also promote stronger polymer–filler interactions by introducing functional groups into the matrix and blocking reactive filler surfaces. In PLA, reactive extrusion with Joncryl has been shown to increase molecular weight and induce long-chain branching, leading to a higher melt viscosity, improved melt strength, and enhanced foamability [39]. In neat PHB and PHB-based composites, Joncryl suppressed chain scission, raised the molecular weight, reduced the melt flow index, and extended the extrusion processing window [40]. In systems containing inorganic fillers, the incorporation of Joncryl ADR-4368F into PLA/talc composites during reactive extrusion reduced degradation and improved the filler–matrix adhesion, resulting in an enhanced mechanical performance and processability [41]. Similar effects were observed in PLA/nanoclay nanocomposites, where Joncryl promoted finer clay dispersion, improved rheological behavior, and enhanced mechanical performance [42]. In systems involving other polymers or biopolymers, phthalic anhydride (PhA) directly incorporated into polylactic acid/poly(butylene adipate-co-terephthalate) (PLA/PBAT) markedly improves phase morphology and overall toughness relative to uncompatibilized blends [43]. Maleic anhydride has also been used in hybrid PLA composites reinforced with short natural fibers and talc, where it improved interfacial adhesion and tensile strength compared to the uncompatibilized system, although its effect was less pronounced than that of multifunctional epoxide chain extenders such as Joncryl^®^ ADR 4368 [41]. In neat PLA, blocked (latent) multifunctional isocyanates have been successfully employed to increase molecular weight and, more importantly, to enhance melt strength: a methanol-blocked polyisocyanate used in the extrusion foaming of PLA enabled the formation of a denser, finer cell structure and markedly improved foam processability, i.e., melt strength and cell morphology [44]. In addition, conventional diisocyanates (particularly hexamethylene diisocyanate (HDI)/methylene diphenyl diisocyanate (MDI)) function as efficient chain extenders in PLA, leading to significant increases in molecular weight (Mw) and stabilization against thermal degradation even in low-molecular-weight prepolymers [45,46]. In PHB matrices (or PHBV as a more processable PHB form), isocyanate chemistry has been applied to link hydroxyl-terminated chains to high-molecular-weight multi-block structures; both urethane and secondary allophanate linkages were reported, increasing molar mass and altering rheology and processability [47]. In PLA/PHBV blends, diisocyanates (HDI, poly(HDI), 1,4-phenylene diisocyanate (PDI)) act as in situ compatibilizers: they refine the phase dispersion, increase the interfacial adhesion, and improve both the mechanical properties and processing windows [48].

Based on the literature review, it can be stated that only a limited number of studies have addressed the application of inorganic fillers in polymer blends based on PLA/PHB. The selection of fillers used in this study was motivated by their availability, the generally recognized lower cost compared to PLA and PHB, and their potential to improve properties, as reported in the literature above, which is of practical importance for extending the application window of PLA/PHB blends. Since the tested fillers may influence the stability of the polymer matrix, three types of modifiers with different mechanisms of action were also included in the study with the aim of further optimizing the properties. The focus was placed on evaluating the potential of these modifiers to enhance thermal stability by preventing chain scission and slowing down degradation. In addition, the modifiers are expected to improve interfacial compatibility, either between the filler and the matrix or between polymer phases.

## 2. Materials and Methods

### 2.1. Materials

**Polymers:** Polylactic acid (PLA) (Luminy^®^ L130, TotalEnergies Corbion, Gorinchem, The Netherlands, ≥99% L-isomer, MFI 10 g/10 min, 190 °C/2.16 kg) and Poly(3-hydroxybutyrate) (PHB) (ENMAT™ Y3000 powder, TianAn Biologic Materials Co., Ltd., Ningbo, China).

**Plasticizer:** CITROFOL^®^ BII (tributyl O-acetylcitrate; Jungbunzlauer Ladenburg GmbH, Ladenburg, Germany).

**Fillers:**

*Aluminum hydroxide*: Alfrimal 104 (Alpha Calcit Füllstoff Gesellschaft mbH & Co. KG, Cologne, Germany, density 2.4 g/cm^3^, specific surface area 10 m^2^/g, average particle size 10 μm) and Alfrimal 106 (Alpha Calcit Füllstoff Gesellschaft mbH & Co. KG, Cologne, Germany, density 2.4 g/cm^3^, specific surface area 7 m^2^/g, average particle size 5.5 μm); *Talc*: HTP3 (IMI Fabi, LLC, Benwood, WV, USA, density 2.8 g/cm^3^, specific surface area 7 m^2^/g, average particle size 17.5 μm), HTP4 (IMI Fabi, LLC, Benwood, WV, USA, density 2.8 g/cm^3^, specific surface area 3.5 m^2^/g, average particle size 30 μm), and HTP05c (IMI Fabi, LLC, Benwood, WV, USA, density 2.8 g/cm^3^, specific surface area 11 m^2^/g, average particle size 7 μm); *Calcium carbonate*: Omyacarb 1T-VA (Omya International AG, production site Vápenná, Czech Republic, specific surface area 4 m^2^/g, average particle size 8 μm) and Calprec PR (Cales de Llierca S.A., Argelaguer, Spain, density 2.7 g/cm^3^, specific surface area 2 m^2^/g, average particle size 0.05 μm); *Magnesium hydroxide*: Securoc B (Sibelco Specialty Minerals Europe, Maastricht, The Netherlands, density 2.4 g/cm^3^, specific surface area 7 m^2^/g, average particle size 2.8 μm), Duhor C-043/S (Duslo a.s., Šaľa, Slovakia, density 2.4 g/cm^3^, specific surface area 6 m^2^/g, average particle size 1.5 μm), and Duhor N-PL/S (Duslo a.s., Šaľa, Slovakia, density 2.4 g/cm^3^, specific surface area 6–10 m^2^/g, average particle size 1.5 μm); *Silica*: Ultrasil 7000GR (Evonik Industries AG, Hanau, Germany, density 2 g/cm^3^, specific surface area 175 m^2^/g, average particle size 0.014 μm).

**Modifiers:** Phthalic anhydride (PhA) (produced by DEZA a.s., Valašské Meziříčí, Czech Republic), IsoQure TT (toluene diisocyanate (TDI) dimer, Acima AG, Buchs, Switzerland), and Joncryl ADR-4368 (multi-functional reactive epoxy-based chain extender, BASF Nederland B.V., Heerenveen, The Netherlands).

### 2.2. Methods

#### 2.2.1. Blend Preparation

All blends were prepared using a laboratory twin screw extruder (Labtech Engineering Co., Ltd., Samutprakarn, Thailand), with a screw diameter of 16 mm and a length-to-diameter (L/D) ratio of 40. The screw geometry incorporated three kneading zones, and atmospheric venting was positioned at the 38 D mark on the barrel. The temperature profile of the device in the direction from the extruder hopper to extruder for all prepared mixtures was as follows: 160 °C, 170 °C, 175 °C, 180 °C, 180 °C, 180 °C, 180 °C, 180 °C, 175 °C, 170 °C. Extrusion was conducted at a screw speed of 140 RPM. The PLA/PHB ratio was 60/40 wt%. The plasticizer content was 9 wt% relative to the polymer blend. The filler was incorporated at 10 vol% into the blend, and the modifier was added at 1 wt% with respect to the blend.

#### 2.2.2. Preparation of Test Films

The performance test films were prepared by attaching a chill-roll line head directly to the twin-screw extruder. The extruder head temperature was 170 °C. The melt emerging from the rectangular cross-sectional die was extruded onto a system of water-cooled rollers and subsequently wound under constant tension to a winding device. Testing specimens for measuring the physical–mechanical properties of the studied polymer mixtures according to ISO 527-3 [49] were prepared from the films. Strips 15 mm wide were cut from the foils by means of a precision cutter.

#### 2.2.3. Rheological Characterization

The processing stability of the prepared polymer blends was evaluated on an RPA 2000 (Alpha Technologies, Hudson, OH, USA) oscillating rheometer using a timed test. The complex viscosity of each sample was measured following a controlled thermo-mechanical loading procedure using an oscillatory rheometer. The specimens were introduced into a biconical test chamber, which was subsequently sealed, and the system temperature was adjusted to 180 °C. A static preheating phase of 0.5 min was applied to ensure complete melting of the samples. Subsequently, oscillatory deformation was imposed at a frequency of 50 cycles per minute (cpm) with an oscillation angle of 30°, corresponding to a shear rate of 22 s^−1^. Under these conditions, the evolution of the complex viscosity was monitored over 10 min. The viscosity value recorded at 5 min was considered a key indicator of processing stability. A lower complex viscosity at this stage was interpreted as indicative of reduced resistance to thermo-mechanical degradation and, consequently, lower processing stability. To eliminate the initial differences in viscosity of various blends, the relative complex viscosity was introduced, defined as follows:

(1) $${Ƞ}_{rel\left(t\right)}^{*}=\frac{{Ƞ}_{\left(t\right)}^{*}}{{Ƞ}_{\left(0\right)}^{*}}$$

where ${Ƞ}_{rel\left(t\right)}^{*}$ is the relative complex viscosity at time *t*, ${Ƞ}_{\left(t\right)}^{*}$ is the complex viscosity at time *t*, and ${Ƞ}_{\left(0\right)}^{*}$ is the complex viscosity at the beginning of the test. 

#### 2.2.4. Thermal Characterization

Differential scanning calorimetry (DSC) measurements were performed using a DSC 1 instrument (Mettler-Toledo Inc., Greifensee, Switzerland). The following thermal program was applied:

1. Isothermal hold at 30 °C for 1 min.
2. First heating from 30 to 190 °C at 10 °C/min.
3. Isothermal hold at 190 °C for 3 min.
4. Cooling from 190 to 30 °C at 10 °C/min.
5. Isothermal hold at 30 °C for 3 min.
6. Second heating from 30 to 190 °C at 10 °C/min.

The prepared sample was placed in the DSC chamber, and heat flow was recorded relative to an empty sealed aluminum pan used as reference, under nitrogen atmosphere (30 mL·min^−1^). Data acquisition and analysis were performed using STARe evaluation software version 16.40 (Mettler-Toledo Inc., Greifensee, Switzerland). The enthalpy values obtained from DSC were recalculated per unit mass of polymer matrix, excluding the filler contribution, unless otherwise stated.

#### 2.2.5. Tensile Test

Tensile tests were carried out on film strips prepared in accordance with ISO 527-3 [49]. For each polymer blend, five specimens were tested. The strips had a width of 15 mm, a gauge distance of 50 mm, and a thickness corresponding to the prepared films. Testing was performed on a universal testing machine (Zwick Roell, Ulm, Germany) equipped with a contact extensometer. The grip separation was set to 50 mm and the crosshead speed to 50 mm·min^−1^. Tensile parameters, including yield stress, tensile strength at break, and elongation at break, were determined from the stress–strain curves.

#### 2.2.6. Scanning Electron Microscopy (SEM)

Morphology of the tested specimens was examined using a JEOL JSM-7500F scanning electron microscope (JEOL Ltd., Tokyo, Japan). For SEM observation, the fracture surfaces of the polymer blend specimens were prepared by cryogenic fracturing in liquid nitrogen in order to obtain brittle fracture surfaces. Powder samples were deposited on double-sided adhesive tape for observation. Prior to observation, the samples were sputter-coated with a thin layer of gold/platinum alloy using a Balzers SCD 050 sputter coater (Balzers AG, Balzers, Liechtenstein).

#### 2.2.7. Fourier Transform Infrared (FTIR) Spectroscopy

ATR-FTIR spectra of the solid-state samples were recorded using a Nicolet 5700 FTIR spectrometer (Thermo Scientific, Madison, WI, USA) in the range of 4000–400 cm^−1^ at room temperature. Each spectrum was collected with a resolution of 4 cm^−1^ by averaging 32 scans, using a diamond ATR crystal.

## 3. Results and Discussion

### 3.1. Rheological Properties

**Figure 1:**
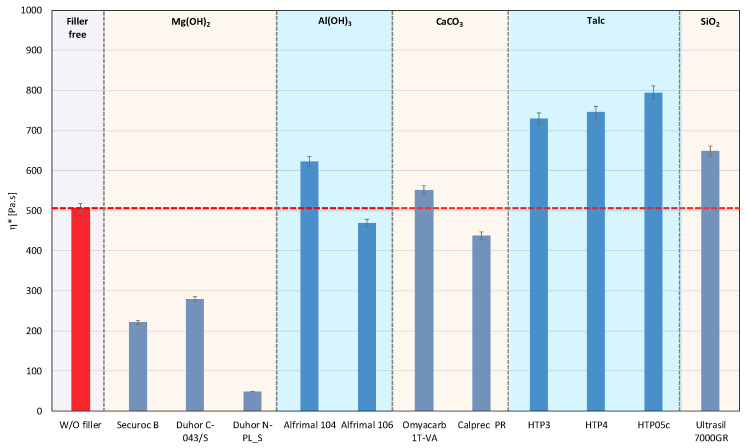
Complex viscosity at the 5th minute of testing for the polymer mixtures with fillers.

**Figure 2:**
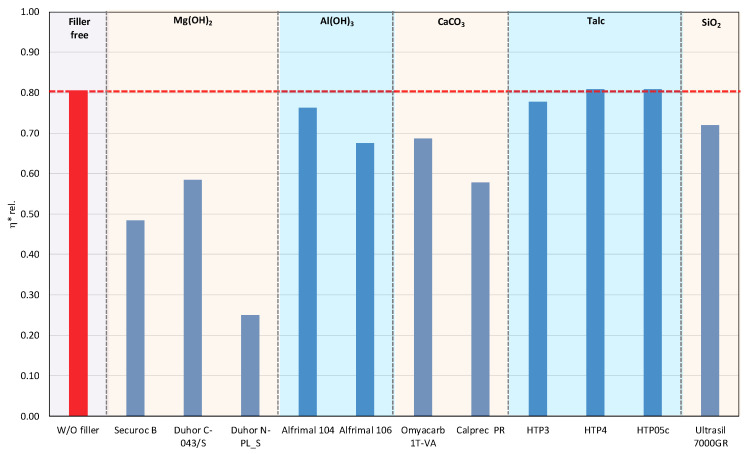
Relative complex viscosity at the 5th minute of testing for the polymer mixtures with fillers.

In polymer systems, the incorporation of fillers usually increases the melt viscosity, particularly when the fillers exhibit a high specific surface area or are surface-modified to promote interfacial compatibility with the polymer matrix, i.e., so-called active or reinforcing fillers. Conversely, the chemical nature of the filler surface may critically affect the processing stability of polymer matrices with a pronounced susceptibility to thermo-mechanical degradation. This is particularly relevant for PLA/PHB blends, which are prone to chain scission and molecular weight reduction under thermo-mechanical stress during melt processing [12,39]. The degradation pathways of these polyesters can be substantially influenced by other blend constituents, including the presence and chemistry of the filler. The processing stability of the prepared mixtures was measured as the dependence of the complex viscosity on time, during which the sample was loaded by shear stress in the oscillating rheometer at 180 °C. The consequence of high temperature and shear stress during the processing of PLA/PHB blends in the melt is the degradation of the material, which causes a decrease in molecular weight following the decrease in viscosity of the mixture. The relative viscosity expresses the change in viscosity during thermomechanical stress over time compared to the flood viscosity value, and thus the degradation of the material itself. For a simple comparison of the influence of individual fillers on processing stability, the values of the viscosities in the 5th minute of the timed test in the oscillating rheometer were evaluated (Figure 1 and Figure 2).

The addition of fillers to the blend led to an increase in viscosity for all tested fillers, except for magnesium hydroxide, one type of calcium carbonate, and one type of aluminum hydroxide, which resulted in a reduction in viscosity compared to the blend without fillers (Figure 1). The reason for the decrease in viscosity from the addition of particular fillers can be either because the fillers are not active or because the filler causes a degradation of the polymeric matrix or both. Considering the results of absolute complex viscosity (Figure 1) in conjunction with the relative complex viscosity evaluation (Figure 2), a conclusion emerges that all types of talc had no pro-degradation influence on the PLA/PHB matrix (relative viscosity after 5 min of melt processing in the rheometer is without changes in comparison to the filler-free blend), and all three tested talcs are active fillers (absolute values of viscosities are higher than the viscosity of the filler-free blend). The highest values of the viscosity among all the tested fillers were achieved with the talc-based mixtures. The HTP05c filler has the smallest particles, and the differences between the talc-based fillers are related to the particle size. Aluminum hydroxide is also an active filler (absolute viscosity of blend with Alfrimal 104 is higher than filler-free blend), but also this type of filler exhibits a moderate pro-degradation effect (slightly lower relative viscosity values). Calcium carbonate Omyacarb 1T-VA also exhibits a similar effect. The final effect on viscosity of the blend containing these fillers is given through the superposition of the reinforcing and degradation effects of fillers. The silica also acts as a pro-degradation substance in the studied PLA/PHB blend (lower relative viscosity), but, similarly to, for example, calcium carbonate Omyacarb 1T-VA, its reinforcing activity is higher; therefore, as a final effect, the viscosity of the blend filled with silica is higher than the unfilled blend. The strongest pro-degradation activity is exhibited by magnesium hydroxide as can be seen in Figure 2, and, due to this strong degradation of the polymeric matrix by the filler addition, the relative as well as the absolute viscosity of the blend is much lower than the viscosity of the unfilled blend. Magnesium hydroxide Duhor N-PL_S exhibits the strongest pro-degradation effect on the PLA/PHB blend, probably due to its very good dispersion in the matrix as will be shown later.

For the processability of PLA/PHB-based polymer composites using various technologies, such as extrusion or injection molding, and to achieve optimal mechanical properties, it will be crucial to minimize or eliminate the potential degradative effects of fillers. The strongest decrease in viscosity was recorded for fillers based on magnesium hydroxide. Thus, it can be stated that the degradation process of PLA/PHB mixtures will be significantly affected by the type of filler used, and especially by its chemical nature. Magnesium hydroxide fillers contain hydroxyl OH groups in their structures, and that is the reason why the mixtures that contain these fillers are the most sensitive to degradation. This conclusion is also supported by the results of work [50], where it has been proven that PHB can undergo an intensive degradation in the presence of two types of alcohol—ethylene glycol and glycerol. Alcoholysis with ethylene glycol is significantly faster than with glycerol, and the results show that degradation proceeds by random chain scission. This confirms that, not only OH presence, but also their reactivity has an influence on the degradation rate of the studied polymeric matrix. For both reactions, there was an observed decrease in the polydispersity index, which means that the degradation was caused by OH groups, which both alcohols contain [50]. An interesting situation is the case of aluminum hydroxide, where the presence of OH groups in this case causes a much lower degradation than magnesium hydroxide. This may be due to the fact that magnesium hydroxide is an ionic compound, while aluminum hydroxide exhibits stronger covalent forces, which may partially prevent the involvement of OH groups in degradation processes reactions.

The degradation of the polymeric matrix, in addition to decreasing the viscosity, can cause also the deterioration of other, mostly mechanical properties of the final material due to the decrease in the molecular weight of polymers created by the matrix.

**Figure 3:**
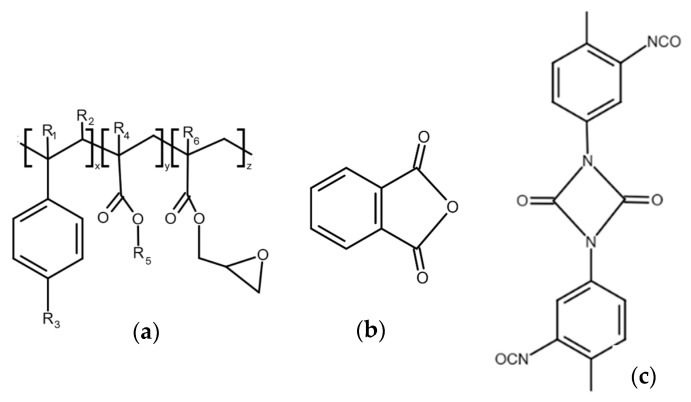
Chemical structure of (**a**) Joncryl 4368, (**b**) phthalic anhydride, and (**c**) IsoQure TT.

**Figure 4:**
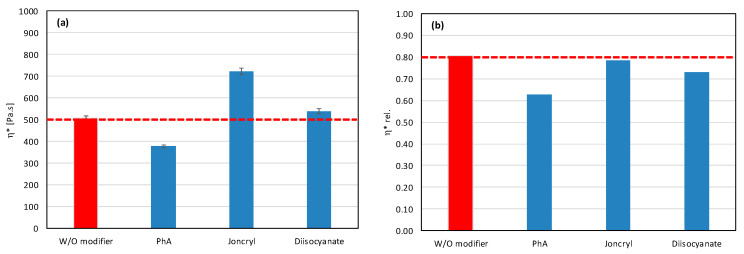
Effect of modifier on (**a**) complex and (**b**) relative complex viscosity at 5 min of testing for matrices without fillers.

In view of these facts, we have tried to eliminate the degradation process by the addition of suitable modifiers. We chose Joncryl (Figure 3a), a multifunctional styrene-acrylic-epoxy-based random oligomer which is the most common chain extender for polyesters including PLA due to its high functionality and chain-extending efficiency by reacting with terminal OH as well as terminal COOH groups of polyester chains [39,40]. We assumed that, in addition, epoxy groups could rapidly react with free OH groups on the filler surface, and, by this, they can protect the polyester against alcoholysis degradation. Phthalic anhydride (PhA) (Figure 3b) and diisocyanate (Figure 3c), which contain anhydride functional group and diisocyanate, respectively, also can rapidly react with OH groups on filler surfaces and, in this way, can protect polymers against the degradation described above. It should also be considered that the highly reactive isocyanate groups are capable of forming durable cross-linked structures, which complicates their straightforward classification as compatibilizers or chain extenders and may strongly affect the melt rheology and processing characteristics [51]. For the identification of the mechanisms of modifier action, it is important to understand how modifiers alone affect the rheological properties of a mixture without fillers; therefore, we measured the complex and relative complex viscosity of mixtures without added fillers. The results are presented in Figure 4.

Only Joncryl has no pro-degradation effect on the polyester matrix, and simultaneously acts as coupling agent (chain extender) (Figure 4). It causes no changes in the relative values of viscosity and increase in the absolute values of viscosity. Both anhydride and diisocyanate act as pro-degradants, but diisocyanate, in contrast to PhA, acts also as a coupling agent. The chemical nature (functional groups) of the modifier can initiate a degradation processes in the polymer matrix itself, so it is very important to find the right combination of filler and modifier, and finally a suitable amount of modifier added to the mixture, to obtain the best balance between all chemical reactions: the degradation of polymers, the endcupping of active OH groups of fillers, and the chain extending of polyesters. Therefore, a basic screening of modifier–filler combinations was realized to find the best option. In the first screening, all modifiers were applied at a concentration of 1 wt%, but it is clear that the quantitative acting of modifiers depends on the molar concentration of active groups in relation to the molar concentration of active OH groups on the filler surface. 

**Figure 5:**
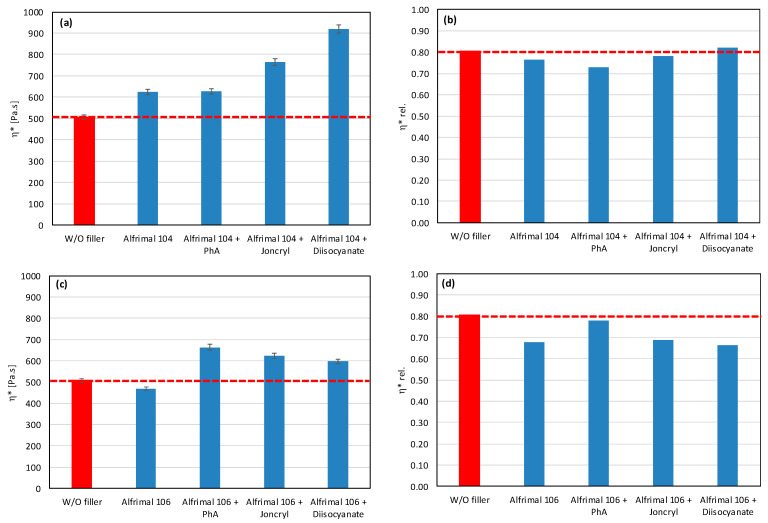
Effect of modifier on complex viscosity (**a**,**c**) and relative complex viscosity (**b**,**d**) at 5 min of testing for the polymer blend containing aluminum hydroxide.

**Figure 6:**
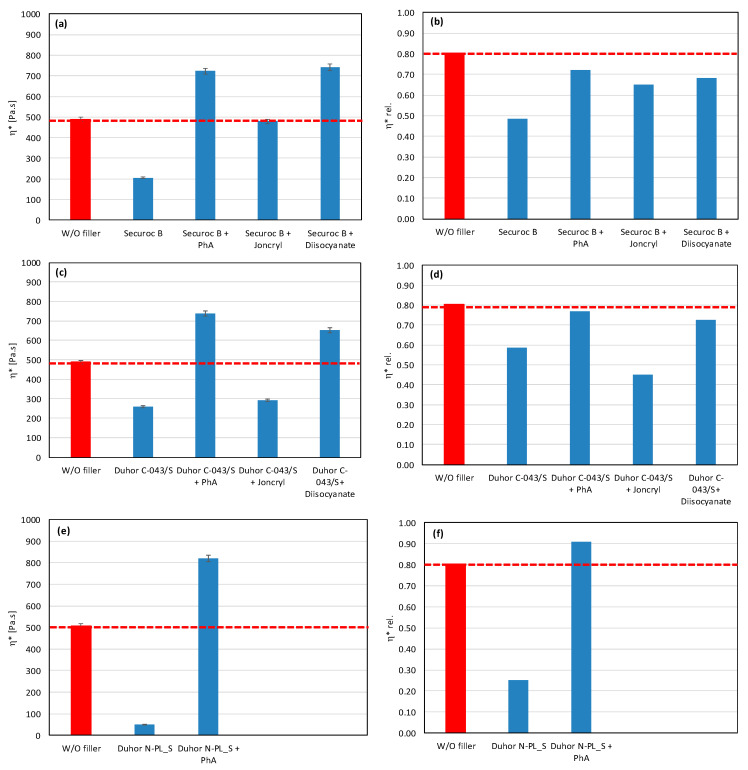
Effect of modifier on complex viscosity (**a**,**c**,**e**) and relative complex viscosity (**b**,**d**,**f**) at 5 min of testing for the polymer blend containing magnesium hydroxide.

As mentioned earlier, fillers with more reactive OH groups (Mg (OH)_2_) have a significantly more pronounced effect on the degradation of the PLA/PHB blends than fillers with less reactive OH groups. Depending on the reactivity of OH groups and the intensity of degradation of the biopolyesters, the degrading effect of the tested potential modifiers is also manifested in various ways. Figure 5 and Figure 6 show the complex viscosity and relative complex viscosity values in the 5th minute of testing for mixtures with aluminum hydroxide and magnesium hydroxide, respectively. Joncryl has practically no or a very weak effect on the processing stabilization of the blends with these two types of fillers (see relative viscosity values). On the other hand, a small increase in the absolute values of viscosity compared to the unmodified blend can be observed as a superposition of the chain extending, reinforcing, and degrading effects, together with the partial elimination of OH groups by a reaction with the epoxy groups. In contrast to Joncryl, more reactive modifiers react more rapidly with fillers’ OH groups than Joncryl, and therefore they exhibit a more effective stabilizing effect. From this point of view, the most effective combination for aluminum hydroxide is Afrimal 104/diisocyanate. This blend has the same relative viscosity value as the unfilled blend and a much higher value of absolute viscosity than the unfilled and filled blends without modifiers. In this case, the modifier ensures a full protection of the matrix against degradation via the OH groups from the filler, and therefore the reinforcing effect of the filler can be developed. The same effect in the case of magnesium hydroxide can be observed in the combination of Duhor C-043/s as a filler and PhA as a modifier. Unmodified magnesium hydroxide (Duhor N-PL_S), which exhibited the strongest pro-degradation effect among all the tested fillers (Figure 1 and Figure 2), was subsequently tested only with the PhA modifier based on the obtained results. Only this combination, out of all the tested filler/modifier alternatives, provides a higher relative viscosity in 5 min than the unfilled mixture, which means that the Duhor N-PL_S/PhA combination has a stabilizing effect on PLA/PHB melting and slows down the degradation rate of the mixture. This is probably due to the high reactivity of the OH groups, which, if unprotected, have a strong pro-degradation effect; on the other hand, in the presence of a bifunctional PhA-type reactive additive, this pro-degradation effect of the free OH groups is partially or fully eliminated.

To provide evidence for the interaction of the modifiers with the surface hydroxyl groups of the fillers, FTIR spectra of magnesium hydroxide and aluminum hydroxide in the presence of the additives were recorded. Dry mixtures were prepared using the same filler-to-modifier ratio as in the studied blends, and were subsequently mixed for five minutes at 180 °C to simulate the conditions of reactive processing. In the O–H stretching region, a strong and sharp absorption band at 3687 cm^−1^ corresponding to the O–H stretching vibration of magnesium hydroxide was observed. The relative intensity of this absorption decreased compared with the pure magnesium hydroxide sample, providing evidence that some of the hydroxyl groups in the mixture samples reacted with the additives, which is reflected in the reduced intensity of the OH absorption signal (Figure S20). For aluminum hydroxide, the O–H stretching region is characterized by several broad absorption bands centered around 3620 cm^−1^, together with a wider envelope extending from approximately 3550 to 3300 cm^−1^. A clear decrease in the intensity of these OH-related bands was observed in the spectra of the mixtures, providing evidence of the interaction of the modifiers with the surface hydroxyl groups of aluminum hydroxide (Figure S19).

**Figure 7:**
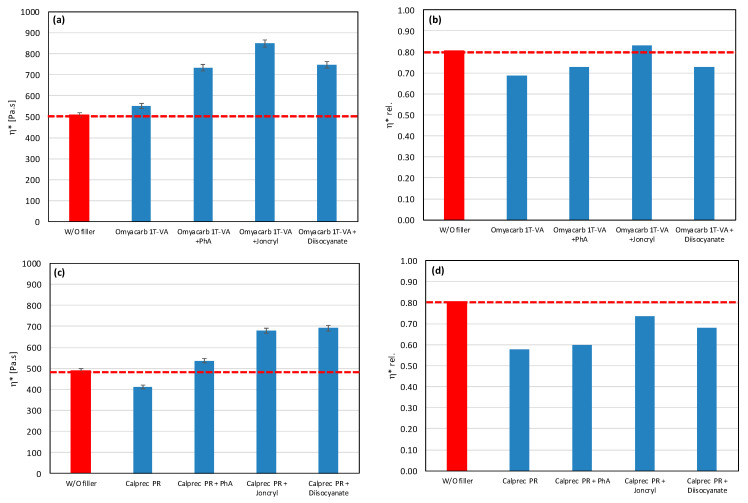
Effect of modifier on complex viscosity (**a**,**c**) and relative complex viscosity (**b**,**d**) at 5 min of testing for the polymer blend containing calcium carbonate.

**Figure 8:**
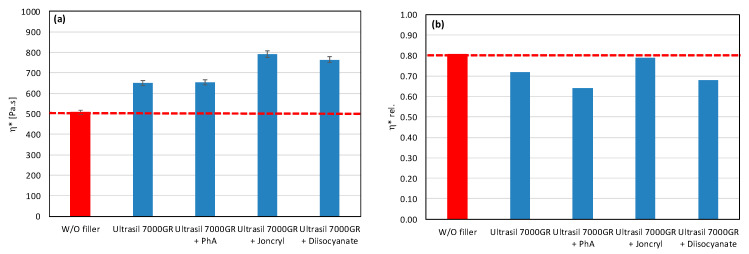
Effect of modifier on complex viscosity (**a**) and relative complex viscosity (**b**) at 5 min of testing for the polymer blend containing silica.

**Figure 9:**
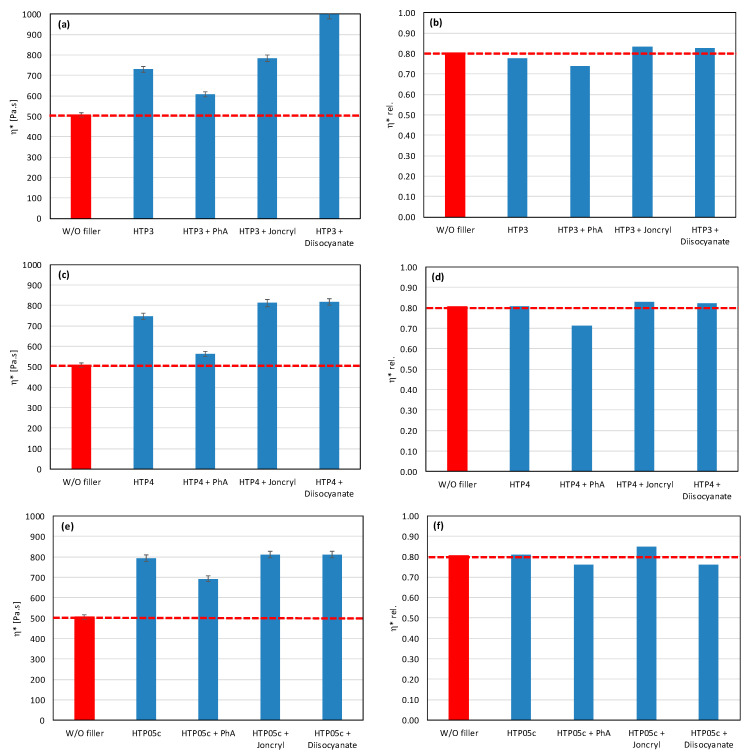
Effect of modifier on complex viscosity (**a**,**c**,**e**) and relative complex viscosity (**b**,**d**,**f**) at 5 min of testing for the polymer blend containing talc.

As a result, even the same type of filler (chemically) may require different types of modifiers to reduce the rate of degradation. All modifiers were tested also in combination with other tested fillers (talc, calcium carbonate, and silica) (Figure 7, Figure 8 and Figure 9). The results confirmed the selectivity of modifiers for protection of the PLA/PHB matrix against processing degradation in the presence of various types of fillers. We could declare that Joncryl exhibited a positive effect in combination with calcium carbonate and silica.

### 3.2. Thermal Properties

**Figure 10:**
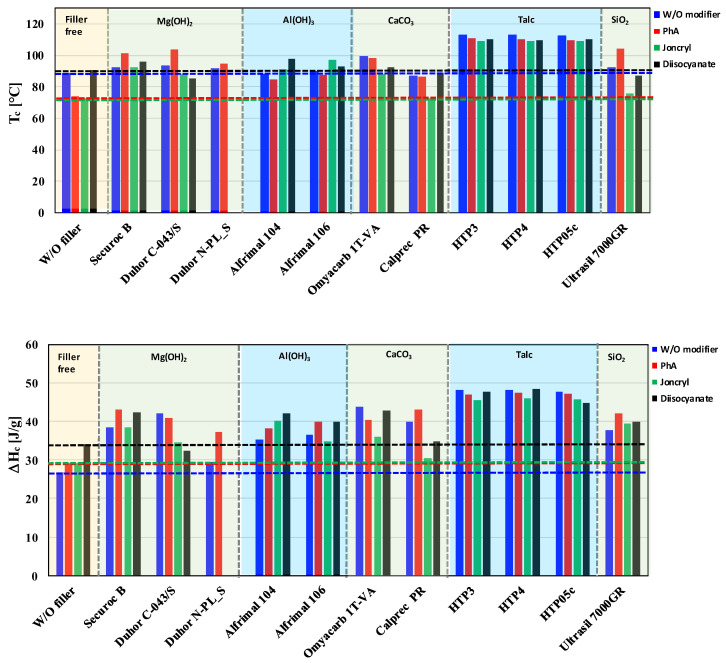
Effect of filler and modifier type on crystallization temperature (T_c_) and enthalpy (ΔH_c_).

Temperature and enthalpy of crystallization are very important parameters for the processing of semicrystalline polymeric materials like PLA/PHB blends. These parameters have a strong influence on the processing and application properties of polymeric materials. A high temperature of crystallization (T_c_) indicates a fast and early crystallization during the cooling of a melt, which allows for a shorter injection molding cycle than in the case of materials with a lower T_c_. The enthalpy of crystallization directly reflects the amount of crystalline phase which is created during the cooling of a melt. Based on DSC thermograms, the crystallization characteristics of the studied blends were evaluated. The influence of fillers and modifiers on the temperature and enthalpy of crystallization is presented in Figure 10. The enthalpy values were recalculated relative to the polymer matrix, thereby eliminating the influence of filler mass and enabling a more accurate comparison of the crystallization behavior between neat and filled systems. 

The crystallization process is significantly affected by the presence of the filler. Talc in particular significantly increases Tc, which in turn allows for a higher content of crystallites in the final product, as documented by the increased enthalpy of crystallization (Figure 10). Talc clearly acts as the most effective nucleating agent among the tested fillers, while the effect of modifiers in the case of talc is negligible. However, as far as fillers that cause a degradation of the polymer matrix are concerned, then the modifiers have a significant effect on the crystallization process. A higher content of the crystalline phase is achieved for those modifiers which at the same time suppress degradation. For OH-functional fillers, these are mainly PhA and diisocyanate. Although Joncryl has a stronger influence on the neat matrix (showing the highest increase in viscosity in the matrix without filler, Figure 4), its modifying effect on either increasing the viscosity or influencing crystallization is usually the lowest. This clearly confirms the correctness of the chosen approach—preventing the degradation of PLA/PHB/inorganic filler composites by modifying the filler surface (blocking pro-degrading OH groups) rather than subsequently reconstructing the degraded polymers by applying coupling agents. As shown in Figure 10, all mixtures with fillers without modifiers show a higher enthalpy of crystallization than the unfilled matrix alone, from which it is clear that the nucleation activity of the fillers is higher than their pro-degradative activity, while the application of suitable modifiers reduces the extent of matrix degradation by fillers containing Mg(OH)_2_ and Al(OH)_3_, subsequently slightly increasing the content of the crystalline phase.

### 3.3. Mechanical Properties

**Figure 11:**
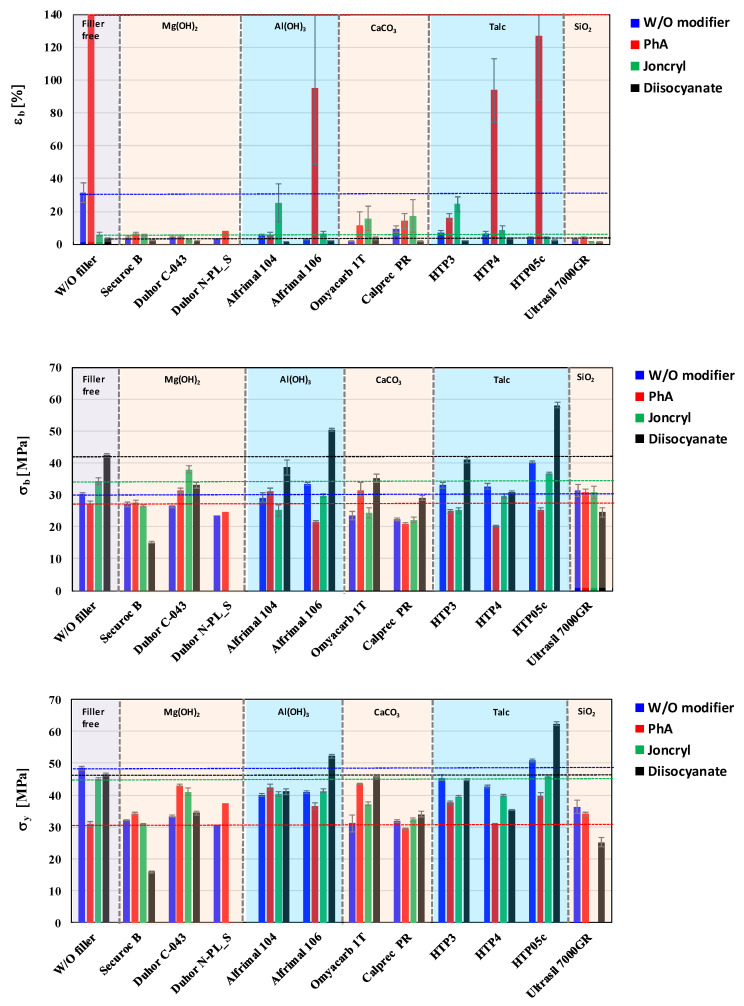
Effect of fillers and modifiers on mechanical properties of PLA/PHB blends.

As part of the assessment of the influence of the type of inorganic filler and modifier on the mechanical properties, in the basic screening part of the work, we used cast foils prepared by chill-roll technology at a lab scale. The relative elongation at break, tensile strength at break, and yield strength were monitored as basic mechanical parameters (Figure 11).

From the point of view of comparing the relative elongation at break for mixtures without modifiers, it can be stated that the highest values were logically reached for the unfilled mixture, at a level of approximately 30%. For filled mixtures without modifiers, the elongation was below 10%. A completely different situation can be observed after the application of the PhA modifier, which interacts with the polymer matrix the most. It can be observed that the relative elongation at break in mixtures without any filler was the highest with the PhA modifier, at approximately 300%. The PhA modifier seems to be the best for mixtures filled with HTP05c talc, HTP4 talc, and Alfirmal 106 as well. With the addition of another modifier (Joncryl), the situation changed completely when an elongation of the unfilled mixture below 10% was recorded; on the contrary, the values for the fillers Alfrimal 104, HTP3, and Omyacarb 1T increased. The changes in the case of extension for mixtures with diisocyanate modifiers are negligible, and the modifying effect has not been confirmed for this property.

It is more complicated to describe the influence of selected fillers and modifiers on the tensile strength at break. The positive effect of the modification on the monitored property can be clearly stated, but its improvement for a specific filler will be given by using the particle size of a filler and its surface treatment by a modifier. In this case, the chemical nature of the filler also plays a role, as the changes for groups of fillers (e.g., talc) are similar. However, in addition to improving the tensile strength at break of the filled mixtures, the selected modifiers also allow an increase in the case of a mixture without a filler. The highest values of tensile strength at break at the level of 58 MPa were achieved with talc (HTP05c), which has the smallest particle size of all types of talc used, in combination with the modifier diisocyanate. Other tested modifiers do not have such a pronounced effect on tensile strength, and their positive or negative effect depends on the type of filler. PhA was most positive in combination with Duhor C-043 and Omyacarb 1T. Joncryl either deteriorated or did not improve tensile strength for most fillers, with the exception of Duhor C-043, where there was a significant improvement. The last property evaluated was the yield strength. From the point of view of assessing the strength characteristics, it will be more important, as the absolute values were achieved higher than in the case of tensile strength at break. As was the case for tensile strength at break, the highest values of yield strength were achieved with HTP05c and Alfrimal 106 filler in combination with the diisocyanate modifier. Based on a summary of the previous considerations, it can be stated that the resulting physical–mechanical properties will be determined not only by the type of filler, its chemical nature, and its particle size, but also by the type of modification. Based on the obtained results, it can be stated that there is no universal modifier to improve the mechanical properties for all types of fillers in the given PLA/PHB matrix. It turned out that, even within one class of fillers, e.g., talc, a given modifier can have a positive effect once and a negative effect depending on the specific properties of the filler, presence of functional groups, their density and reactivity, and also the specific surface area and the size of the particles.

### 3.4. Morphological Structure

**Figure 12:**
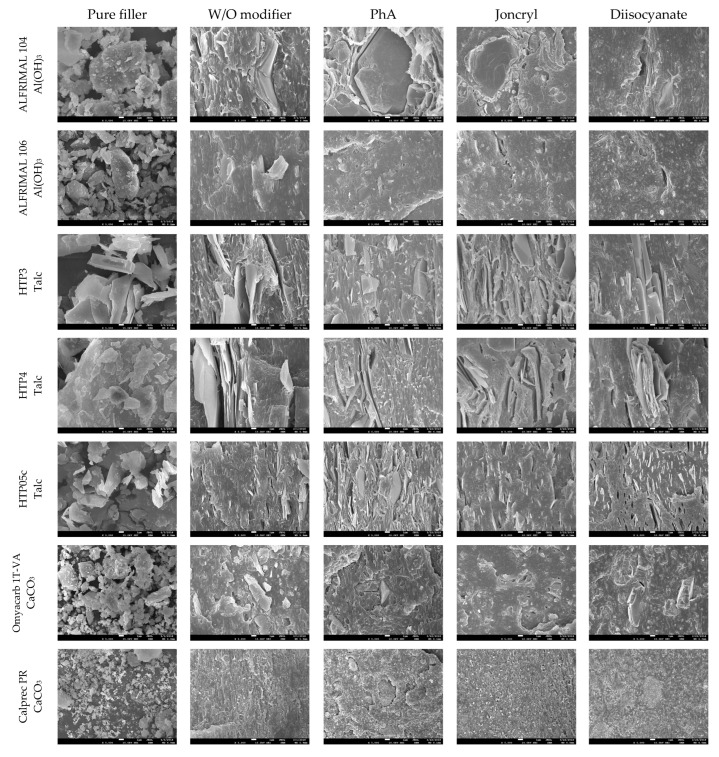

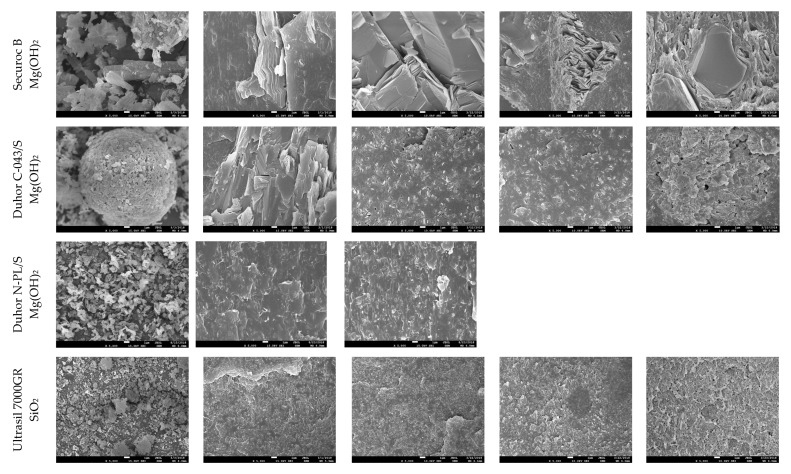
SEM images of the morphology of filled blends at 5000× magnification.

Most properties, preferably mechanical, are closely related to the morphological structure of the blends. Important factors in this regard are the dispergation of the filler and the phase interface between the filler and the polymer matrix. A morphological study was conducted based on SEM images of fracture surfaces prepared in liquid nitrogen. SEM images at 5000× magnification are shown in Figure 12. The tested modifiers, regardless of their antidegradative activity in the mixture, have different effects on their morphologies.

A comparison of the two types of aluminum hydroxide shows that Alfrimal 106 has smaller filler particles compared to Alfrimal 104. In the process of shear stress on a twin-screw extruder, in the case of the filler Alfrimal 104, the filler did not break into smaller particles; it can also be seen that, in the case of Alfrimal 104, after mixing into the mixture, the compatibility at the phase interface is worse than in the case of Alfrimal 106. At the same time, it can be seen that the addition of the modifier Joncryl and diisocyanate to the Afrimal 104 improved the compatibility between the filler and the polymer matrix at the phase interface in comparison to PhA, which corresponds to the following physical–mechanical properties: the highest elongation values for the modifier Joncryl and the highest values of tensile strength for the modifier diisocyanate. Smaller filler particles in the case of Alfrimal 106 and their better dispersion in the polymer matrix were responsible for a further increase in physical–mechanical properties compared to Alfrimal 104. In general, both tested aluminum hydroxide fillers have more potential as a filler for PLA/PHB blends with Afrimal 106, and, based on the morphology of the blends, the best results use PhA as a modifier. This also corresponds with mechanical properties, and this modifier offers the best processing stability of filled blends with Afrimal 106.

Talc-based fillers have a lamellar structure, where the decisive factor will be the ability to delaminate in the shear field during the blending. This effect is most pronounced in the case of talc HTP05c, and especially when applying the modifiers Joncryl and diisocyanate. This correlates with the obtained physical–mechanical properties, as the highest values of tensile strength at yield and tensile strength at break were achieved for all tested fillers. The other two types of talc (HTP3 and HTP4) show a significantly lower degree of delamination in the mixing process than HTP05c talc, regardless of the modifier applied.

If calcium carbonate, under the trade name Omyacarb 1T, was used as a filler, an improvement in compatibility with the polymer matrix could be observed on the fracture surfaces as a result of the applied modifiers. In the case of the PhA and diisocyanate modifiers, this was reflected in higher values of tensile strength at break. A relatively wide particle size distribution can be observed in the images for the pure calcium carbonate filler Calprec PR. On the fracture surfaces of polymer mixtures, larger particles can be observed with the modifiers PhA and diisocyanate. Their comparison shows that a much better compatibility with the matrix is ensured by the addition of the diisocyanate modifier. On the other hand, Omyacarb particles, in general, are significantly larger than Calprec PR particles, which gives Calprec PR a greater application potential in PLA/PHB mixtures, but, compared to other types of fillers tested, mixtures tested with calcium carbonate can be assessed as mildly mixed.

In the case of magnesium hydroxide fillers (Securoc B, Duhor C-043 and Duhor N-PL_S), the modification was carried out mainly to suppress the degradation process during thermomechanical stress. Microscopy images show that there is a difference in the size and shape of the particles of these fillers. Securoc B has a much larger particle size compared to both Duhors, but, in the case of Duhor C-043, it is possible to observe relatively large spherical agglomerates of small elementary particles. Duhor N-PL_S is in the form of significantly smaller agglomerates than Duhor C-043/S. After mixing all three fillers into the mixture without modifiers, Duhor N-PL_S disperses very well; there is no significant dispersion of Duhor C-043/S, and the fracture surface structure contains relatively large areas of non-dispersed magnesium hydroxide. In the case of Securoc B, relatively large compact particles are present in the fracture area. Even the addition of modifiers does not result in a significant reduction in the Securoc B filler particle size, although PhA and Joncryl in particular visibly improve interfacial adhesion, resulting in the destruction of the filler particles themselves rather than delamination onto the particle surface during fracture. On the other hand, in the case of Duhor C-043/S, it can be seen that the modifiers, in particular PhA and Joncryl, ensure a very good dispersion of this filler down to the isolated primary particles. The diisocyanate has a significantly weaker dispersing efficiency, although it also improves adhesion at the phase interface. A good dispersion due to these modifiers probably also occurs due to the reaction between OH groups on the particle surface, which reduces the number of strong OH-OH interactions between the filler particles, which allows them to be more easily dispersed in a relatively low-viscosity less-polar PLA/PHB matrix. Duhor N-PL_S, as already mentioned, is very well dispersed, even without a modifier, and its dispersibility does not change with the addition of PhA. Also, due to the very good dispersibility and thus due to the large surface area of the filler available for the polymer matrix, the PLA/PHB mixture is highly sensitive to degradation due to this type of unmodified Duhor N-PL_S. After the elimination of OH groups through a reaction with PhA, the stability of the mixture is much better; even the relative viscosity, as previously described, is higher than the relative viscosity of the unfilled mixture, which means that this filler has a reinforcing effect in terms of the viscosity of the mixture. For these reasons, magnesium hydroxide Duhor N-PL_S, modified with PhA, seems to be the most promising filler for PLA/PHB mixtures.

The last monitored filler was silica, under the trade name Ultrasil 7000GR. This filler should have the smallest particle size and thus the largest specific surface area, since we expected that it could achieve good values for physical–mechanical properties. In addition, the observation of the morphological structure shows that the filler was very well dispersed in the polymer matrix during the mixing process, but only in small agglomerates, not as primary particles. The dimensions of the agglomerates depend on the applied modifier. However, on the fracture surfaces for modifier Joncryl and, in particular, modifier diisocyanate, a worse compatibilization effect of the modifier can be observed, which would indicate a poorer adhesion to the polymer matrix. A problem with this filler may be the tendency to form agglomerates, which can be seen in the images for the pure filler as well as for the mixtures with the modifiers Joncryl and diisocyanate.

Based on the morphology evaluation, it can be stated that improving the compatibility between the filler and the polymer matrix can be achieved by adding modifiers, but the effect of a particular modifier will have to be assessed in relation to a particular filler, and it is not possible to generalize the effect of modifiers even within fillers of the same chemical nature.

## 4. Conclusions

The main objective of this work was to investigate the potential of applying inorganic fillers to bio-based and biodegradable PLA/PHB blends. For this purpose, commonly used commercial fillers were employed—calcium carbonate, talc, silica, magnesium hydroxide, and aluminum hydroxide—with different particle sizes and particle size distributions. In addition to these fillers, three types of modifiers (PhA, Joncryl ADR-4368, IsoQure TT diisocyanate) were also investigated to further adjust the material characteristics and enhance the overall performance of the blends.

Fillers containing reactive hydroxyl groups on their surface act as pronounced pro-degradants in PLA/PHB blends according to the executed rheological measurements. The results indicate that magnesium hydroxide exerts a stronger pro-degradation effect on the polymer matrix than aluminum hydroxide. The pro-degradative effect of fillers can be suppressed by introducing reactive modifiers capable of binding to the active hydroxyl groups on the filler surface during blend preparation, thereby preventing the degradation of PLA and PHB polyesters. The FTIR spectra of magnesium hydroxide and aluminum hydroxide mixed with modifiers indicate the interaction of modifiers with the surface hydroxyl groups of the fillers, which is reflected in the reduced intensity of the characteristic O–H absorption bands. The results also demonstrated that the efficiency of individual modifiers is highly specific to the type of filler, necessitating the selection of an appropriate filler/modifier combination not only between chemically distinct fillers but also among fillers of the same chemical nature with different morphologies. Among the tested alternatives, the most effective combinations were identified as magnesium hydroxide with phthalic anhydride and talc with diisocyanate. The crystallization behavior of PLA/PHB blends was strongly influenced by the presence of fillers, with talc acting as the most effective nucleating agent, significantly increasing Tc and the enthalpy of crystallization, while the effect of modifiers in this case was negligible. In contrast, for OH functional fillers such as Mg(OH)_2_ and Al(OH)_3_, modifiers played a decisive role, as PhA and diisocyanate suppressed matrix degradation and thereby promoted a higher crystallinity. The study confirmed that the effect of modifiers on the mechanical properties of PLA/PHB blends strongly depends on the type and morphology of the filler. While PhA significantly improved elongation at break, particularly in unfilled blends and in mixtures with talc HTP05c, Alfrimal 106, and HTP4, Joncryl enhanced this property only for Alfrimal 104, HTP3, and Omyacarb 1T, but reduced it in unfilled blends. In contrast, diisocyanate modifiers had a negligible effect on elongation at break, although they proved to be the most effective in enhancing tensile and yield strength in combination with talc HTP05c and Alfrimal 106. The morphological analysis revealed that the effectiveness of the modifiers strongly depends on the specific filler type: for some fillers, they enhance particle dispersion and interfacial adhesion, resulting in improved mechanical properties, while for others their effect is limited.

## Data Availability

The original contributions presented in this study are included in the article/supplementary material. Further inquiries can be directed to the corresponding author.

## Supplementary Materials

The following supporting information can be downloaded at https://www.mdpi.com/article/10.3390/polym17202721/s1. Figure S1. Dependence of complex viscosity on time for the tested mixtures containing different fillers; Figure S2. Dependence of relative complex viscosity on time for the tested mixtures containing different fillers; Figure S3. Dependence of complex viscosity (A) and relative complex viscosity (B) on time for the tested mixtures containing different modifiers; Figure S4. Dependence of complex viscosity (A) and relative complex viscosity (B) on time for the tested mixtures containing Securoc B (Mg(OH)_2_) filler with different modifiers; Figure S5. Dependence of complex viscosity (A) and relative complex viscosity (B) on time for the tested mixtures containing Duhor C-043/S (Mg(OH)_2_) filler with different modifiers; Figure S6. Dependence of complex viscosity (A) and relative complex viscosity (B) on time for the tested mixtures containing Alfrimal 104 (Al(OH)_3_) filler with different modifiers; Figure S7. Dependence of complex viscosity (A) and relative complex viscosity (B) on time for the tested mixtures containing Alfrimal 106 (Al(OH)_3_) filler with different modifiers; Figure S8. Dependence of complex viscosity (A) and relative complex viscosity (B) on time for the tested mixtures containing Omyacarb 1T VA (CaCO_3_) filler with different modifiers; Figure S9. Dependence of complex viscosity (A) and relative complex viscosity (B) on time for the tested mixtures containing Calprec PR (CaCO_3_) filler with different modifiers; Figure S10. Dependence of complex viscosity (A) and relative complex viscosity (B) on time for the tested mixtures containing HTP3 (Talc) filler with different modifiers; Figure S11. Dependence of complex viscosity (A) and relative complex viscosity (B) on time for the tested mixtures containing HTP4 (Talc) filler with different modifiers; Figure S12. Dependence of complex viscosity (A) and relative complex viscosity (B) on time for the tested mixtures containing HTP05c (Talc) filler with different modifiers; Figure S13. Dependence of complex viscosity (A) and relative complex viscosity (B) on time for the tested mixtures containing Ultrasil 7000GR (SiO_2_) filler with different modifiers; Figure S14. Heat flow as a function of temperature for the pure polymers (PLA and PHB); Figure S15. Heat flow as a function of temperature for mixtures with all fillers without modifier; Figure S16. Heat flow as a function of temperature for mixtures with all fillers with modifier PhA; Figure S17. Heat flow as a function of temperature for mixtures with all fillers with modifier Joncryl; Figure S18. Heat flow as a function of temperature for mixtures with all fillers with modifier diisocyanate; Table S1. Results of *t*-tests comparing mechanical properties (elongation at break, tensile strength at break, tensile stress at yield) of PLA/PHB composites with different fillers without and with modifiers (PhA, Joncryl, Diisocyanate); Figure S19. FTIR spectra of Alfrimal 104 (Al(OH)_3_) powder and its mixtures with PhA, Joncryl, and diisocyanate after thermal treatment at 180 °C for 5 min; Figure S20. FTIR spectra of Duhor N-PL/S (Mg(OH)_2_) powder and its mixtures with PhA, Joncryl, and diisocyanate after thermal treatment at 180 °C for 5 min.
