# Supplementary material for: Biodegradable PLA/PHB Composites with Inorganic Fillers and Modifiers
Source: Polymers (Basel). 2025 Oct 10;17(20):2721. doi: 10.3390/polym17202721 (PMC12566949; doi:10.3390/polym17202721)
Supplement: Supplementary file 1 [file polymers-17-02721-s001.zip › polymers-3877288-supplementary.pdf]

Dependence of complex viscosity on time for the tested mixtures containing different fillers is shown in Figure S1. Dependence of relative complex viscosity on time for the tested mixtures containing different fillers is show in Figure S2.

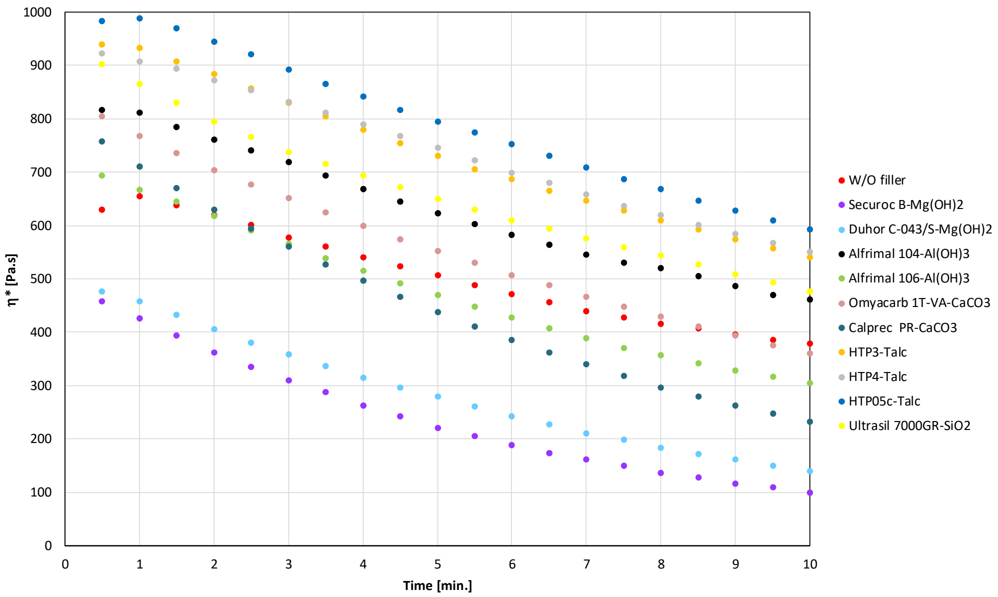

Figure S1. Dependence of complex viscosity on time for the tested mixtures containing different fillers.

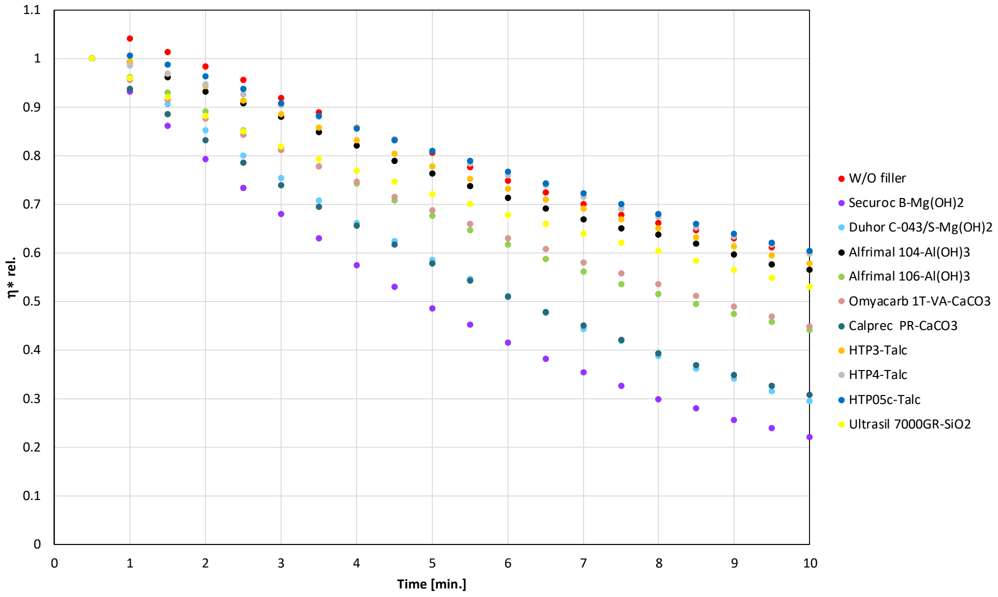

Figure S2. Dependence of relative complex viscosity on time for the tested mixtures containing different fillers.

Dependencies of complex and relative complex viscosity on time (processing stability measurement) for the samples containing different types of reactive modifiers (Figure S3) and same dependencies for samples containig various types of studied fillers and modifiers (Fig. S4 – Fig. S13).

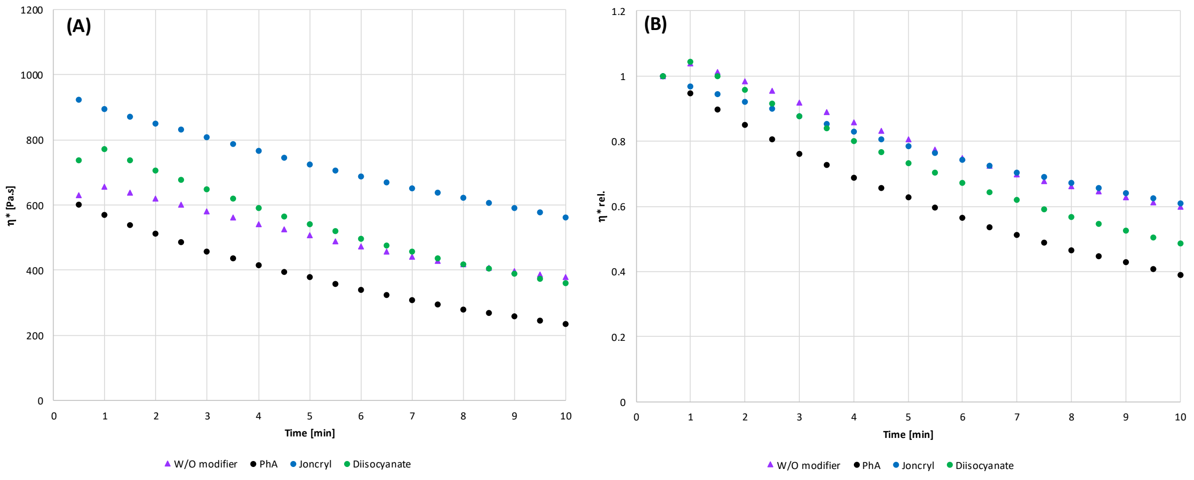

Figure S3. Dependence of complex viscosity (A) and relative complex viscosity (B) on time for the tested mixtures containing different modifiers.

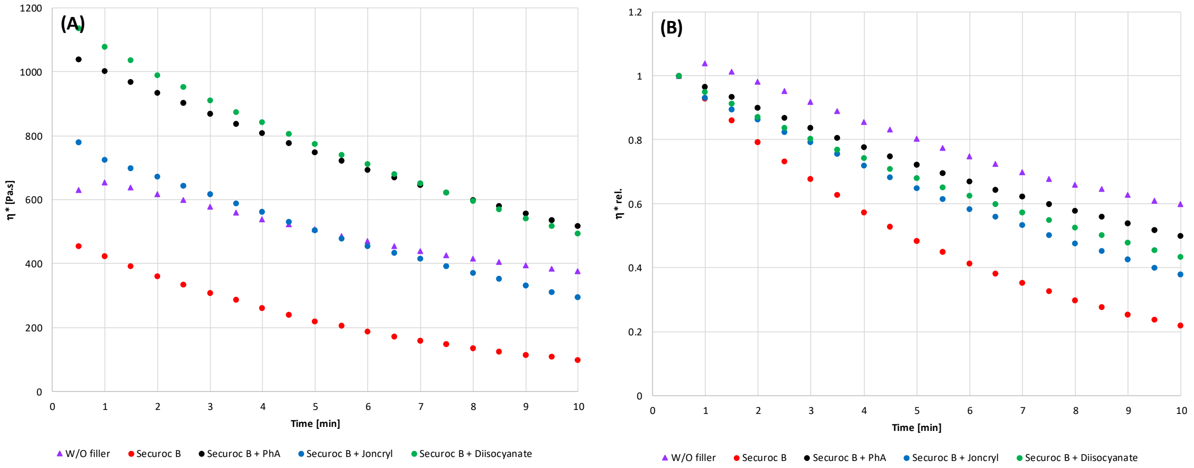

Figure S4. Dependence of complex viscosity (A) and relative complex viscosity (B) on time for the tested mixtures containing Securoc B ($Mg(OH)_2$) filler with different modifiers.

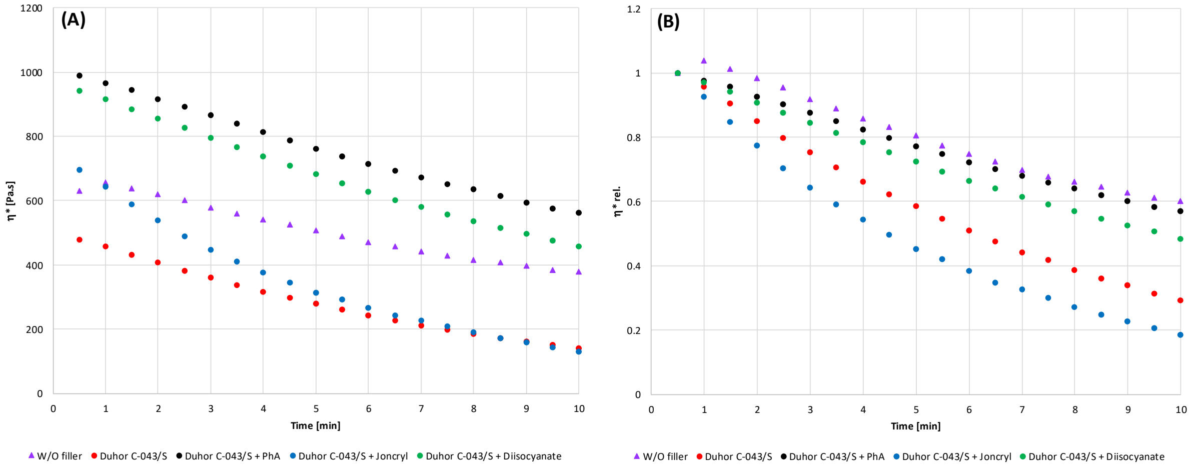

Figure S5. Dependence of complex viscosity (A) and relative complex viscosity (B) on time for the tested mixtures containing Duhor C-043/S ($Mg(OH)_2$) filler with different modifiers.

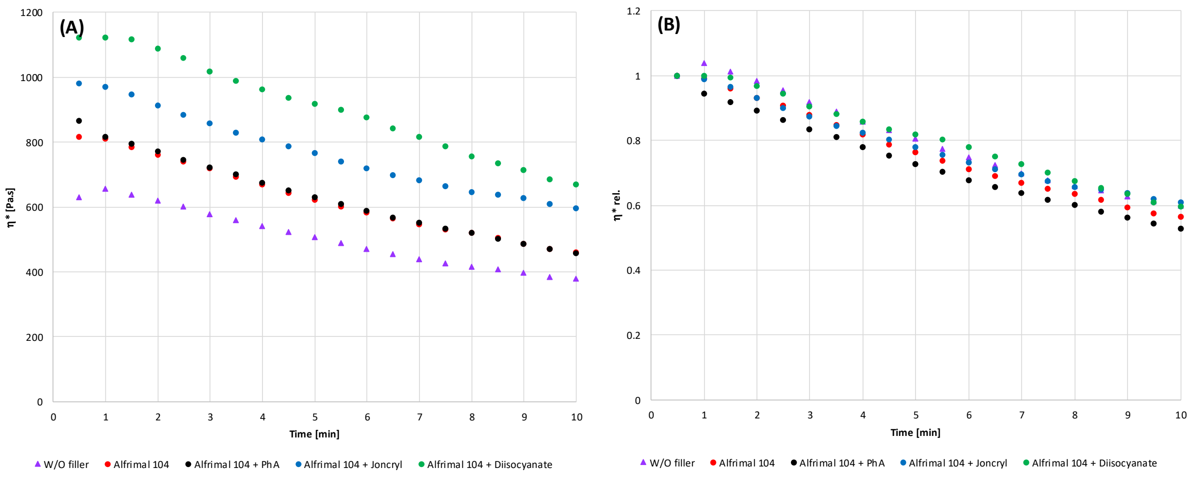

Figure S6. Dependence of complex viscosity (A) and relative complex viscosity (B) on time for the tested mixtures containing Alfrimal 104 ($Al(OH)_3$) filler with different modifiers.

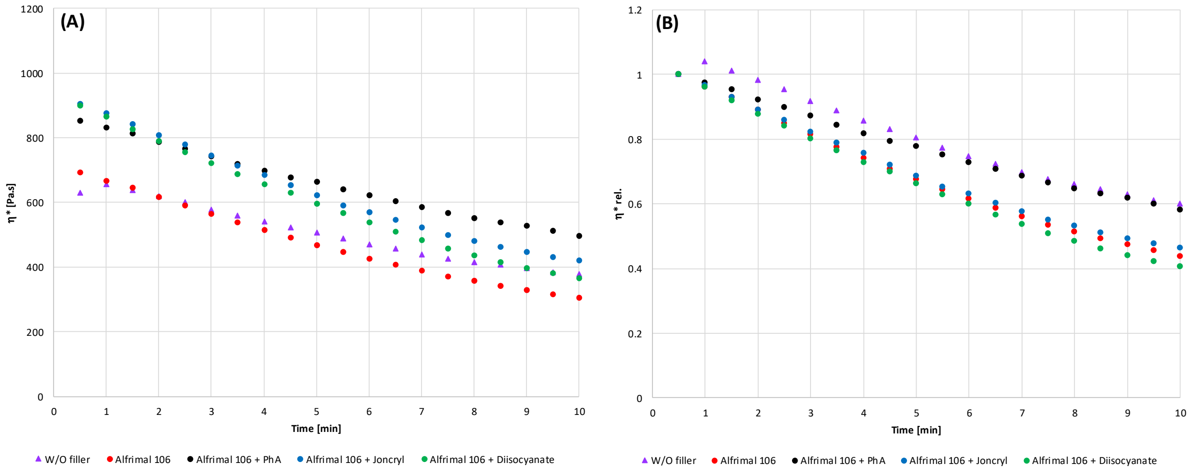

Figure S7. Dependence of complex viscosity (A) and relative complex viscosity (B) on time for the tested mixtures containing Alfrimal 106 ($Al(OH)_3$) filler with different modifiers.

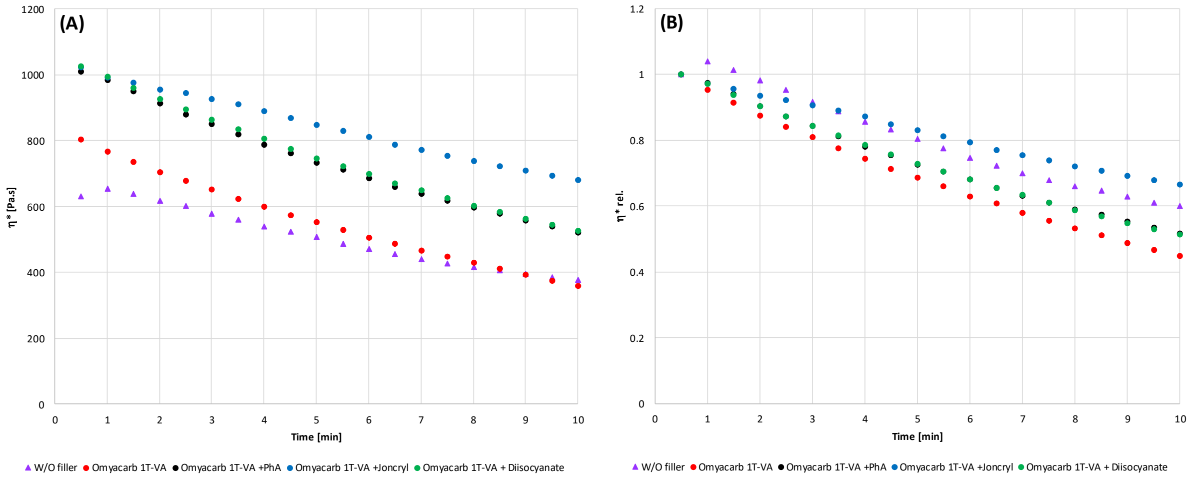

Figure S8. Dependence of complex viscosity (A) and relative complex viscosity (B) on time for the tested mixtures containing Omyacarb 1T VA ($CaCO_3$) filler with different modifiers.

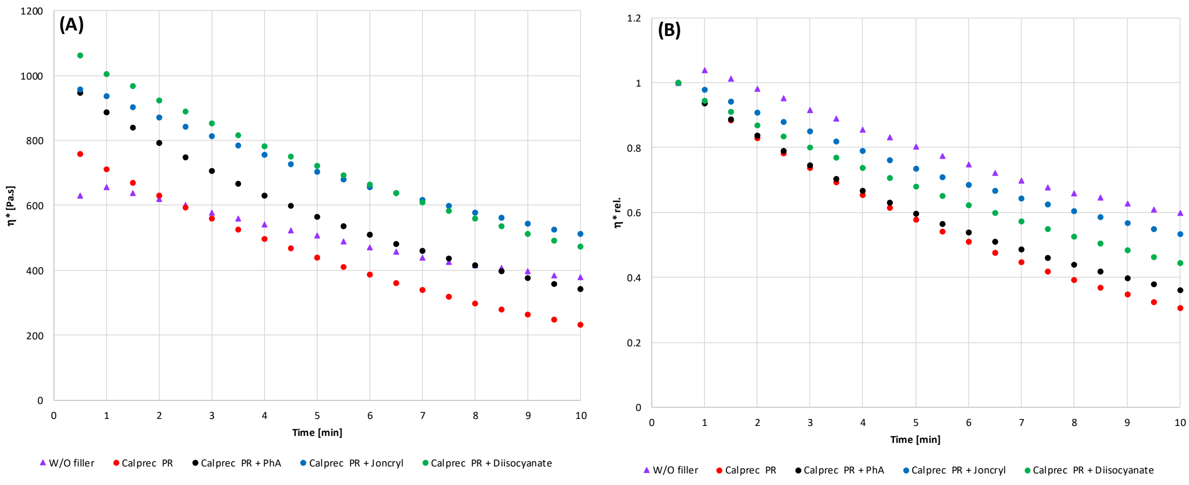

Figure S9. Dependence of complex viscosity (A) and relative complex viscosity (B) on time for the tested mixtures containing Calprec PR ($CaCO_3$) filler with different modifiers.

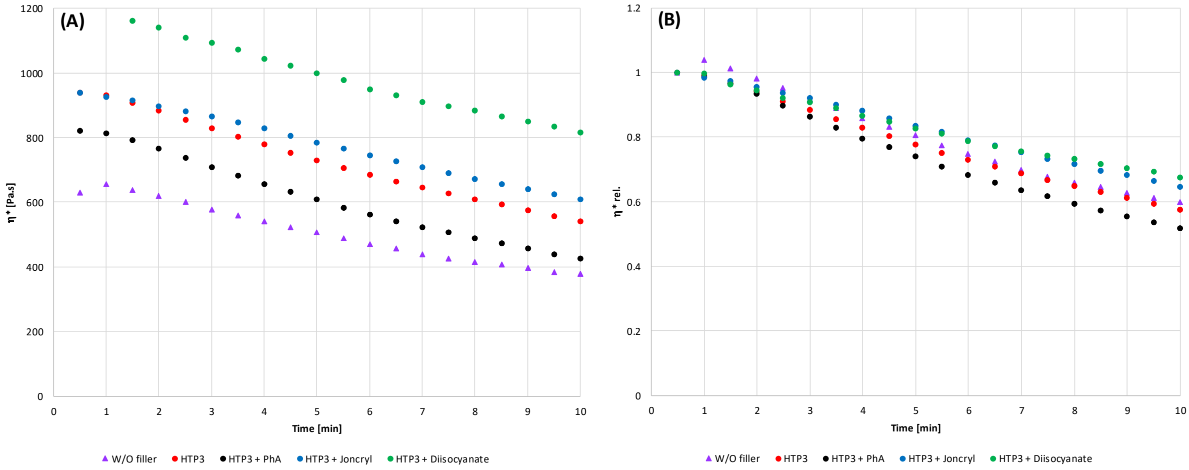

Figure S10. Dependence of complex viscosity (A) and relative complex viscosity (B) on time for the tested mixtures containing HTP3 (Talc) filler with different modifiers.

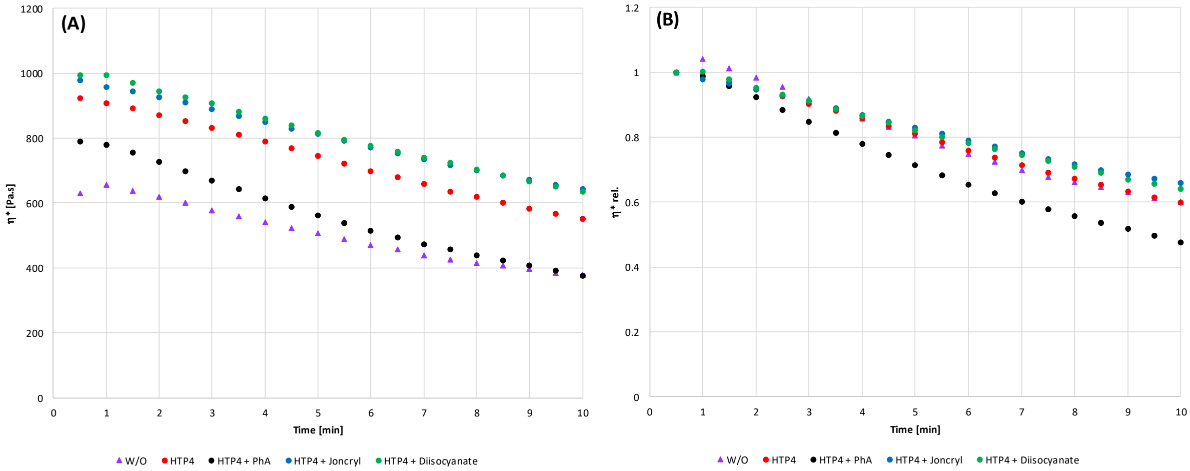

Figure S11. Dependence of complex viscosity (A) and relative complex viscosity (B) on time for the tested mixtures containing HTP4 (Talc) filler with different modifiers.

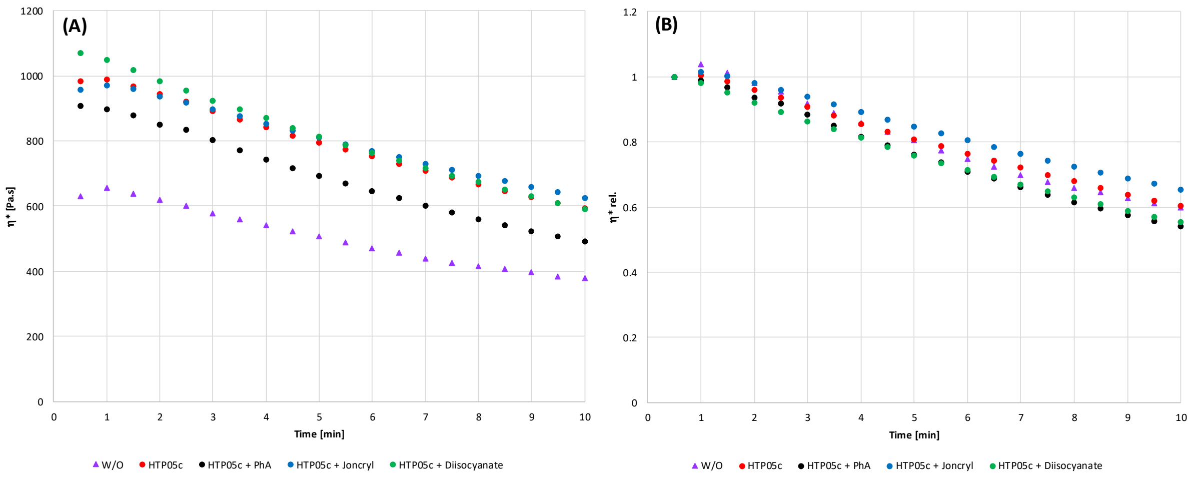

Figure S12. Dependence of complex viscosity (A) and relative complex viscosity (B) on time for the tested mixtures containing HTP05c (Talc) filler with different modifiers.

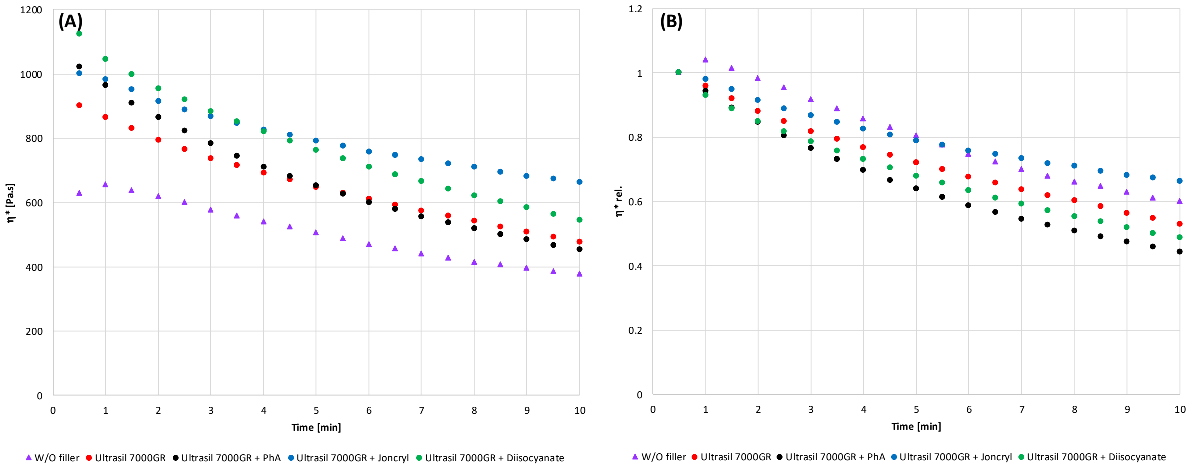

Figure S13. Dependence of complex viscosity (A) and relative complex viscosity (B) on time for the tested mixtures containing Ultrasil 7000GR (SiO_2) filler with different modifiers.

The thermograms of the studied samples were moved in order to shorten the Experimental section. In the manuscript, only the graphs of the described thermophysical properties are included.

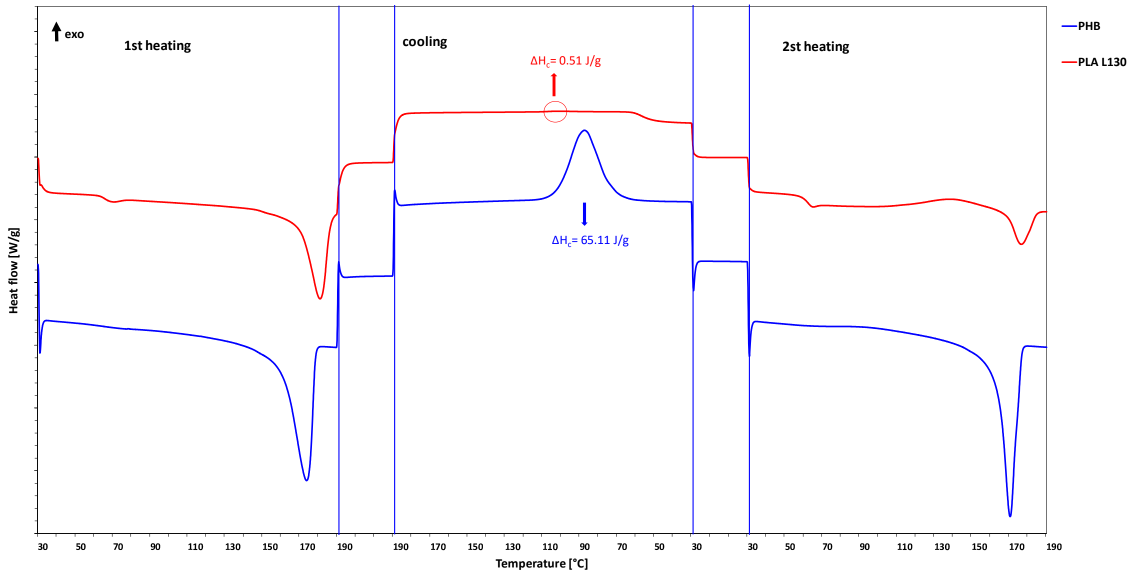

Figure S14. Heat flow as a function of temperature for the pure polymers (PLA and PHB).

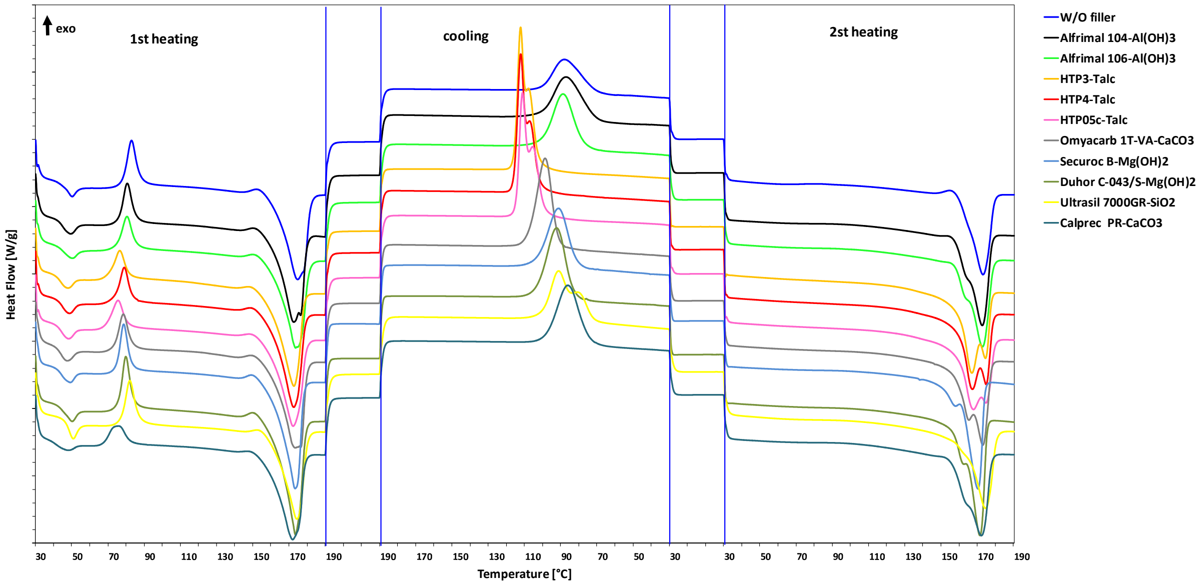

Figure S15. Heat flow as a function of temperature for mixtures with all fillers without modifier.

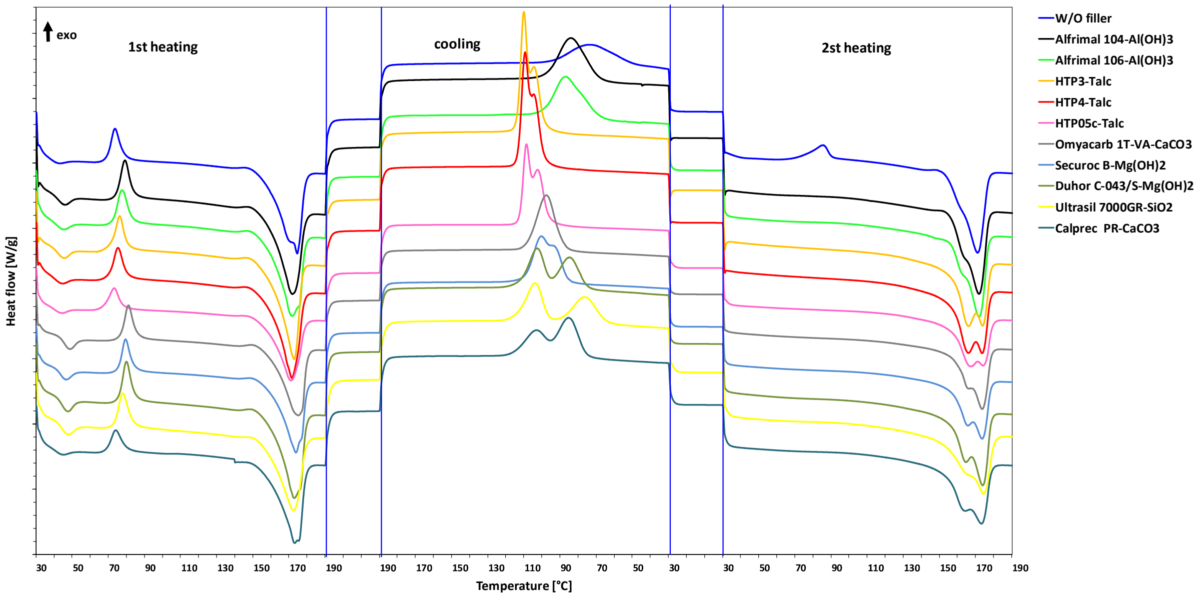

Figure S16. Heat flow as a function of temperature for mixtures with all fillers with modifier PhA.

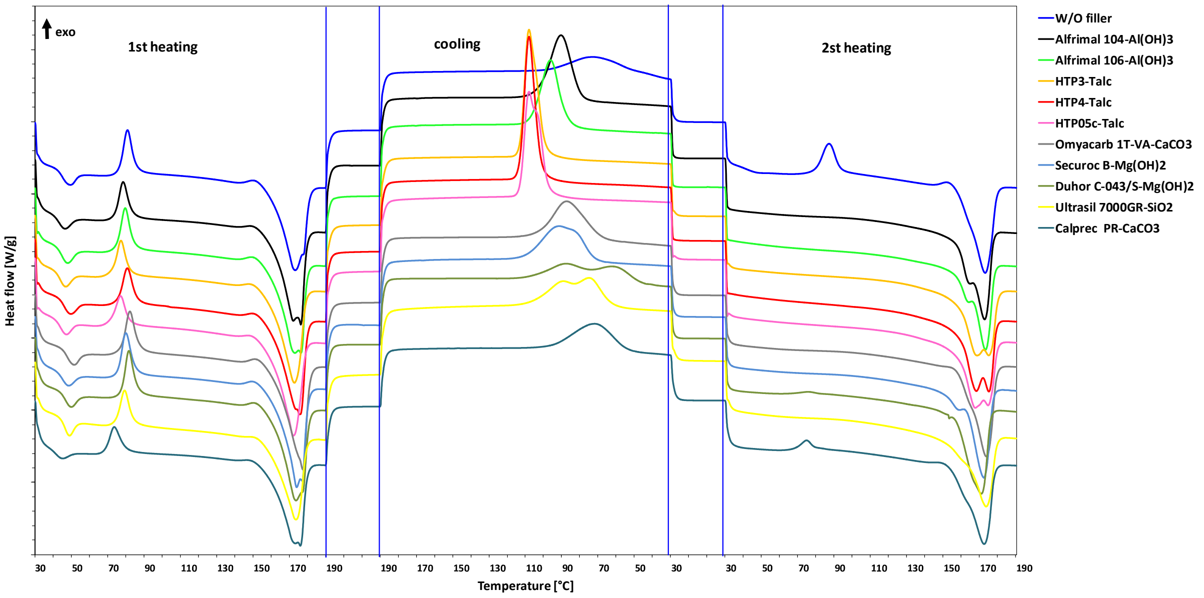

Figure S17. Heat flow as a function of temperature for mixtures with all fillers with modifier Joncryl.

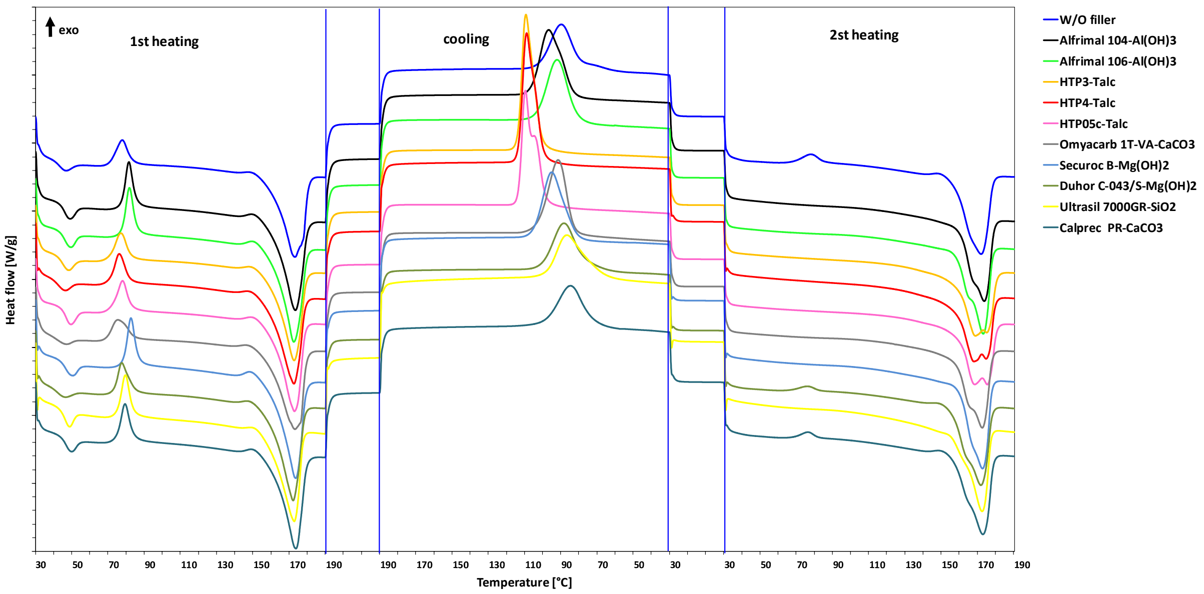

Figure S18. Heat flow as a function of temperature for mixtures with all fillers with modifier diisocyanate.

Statistical evaluation of mechanical properties

Table S1. Results of t-tests comparing mechanical properties (elongation at break, tensile strength at break, tensile stress at yield) of PLA/PHB composites with different fillers without and with modifiers (PhA, Joncryl, Diisocyanate).

Filler	Property	Compared groups	p value	Significance
W/O filler	Elongation at break [%]	W/O modifier vs. with PhA	1.72×10^{-10}	***
		W/O modifier vs. with Joncryl	0.012	*
		W/O modifier vs. with Diisocyanate	0.010	**
	Tensile strength at break [Mpa]	W/O modifier vs. with PhA	0.007	**
		W/O modifier vs. with Joncryl	0.007	**
		W/O modifier vs. with Diisocyanate	4.93×10^{-8}	***
	Tensile stress at yield [MPa]	W/O modifier vs. with PhA	8.70×10^{-8}	***
		W/O modifier vs. with Joncryl	0.002	**
		W/O modifier vs. with Diisocyanate	0.028	*
Securoc B $Mg(OH)_2$	Elongation at break [%]	W/O modifier vs. with PhA	0.058	ns
		W/O modifier vs. with Joncryl	0.244	ns
		W/O modifier vs. with Diisocyanate	0.044	*
	Tensile strength at break [Mpa]	W/O modifier vs. with PhA	0.482	ns
		W/O modifier vs. with Joncryl	0.531	ns
		W/O modifier vs. with Diisocyanate	2.01×10^{-7}	***
	Tensile stress at yield [MPa]	W/O modifier vs. with PhA	0.012	*
		W/O modifier vs. with Joncryl	1.36×10^{-4}	***
		W/O modifier vs. with Diisocyanate	2.91×10^{-11}	***

Duhor C-043/S $Mg(OH)_2$	Elongation at break [%]	W/O modifier vs. with PhA	0.847	ns
		W/O modifier vs. with Joncryl	0.011	*
		W/O modifier vs. with Diisocyanate	0.006	**
	Tensile strength at break [Mpa]	W/O modifier vs. with PhA	3.20×10^{-4}	***
		W/O modifier vs. with Joncryl	1.13×10^{-4}	***
		W/O modifier vs. with Diisocyanate	0.001	***
	Tensile stress at yield [MPa]	W/O modifier vs. with PhA	8.80×10^{-8}	***
		W/O modifier vs. with Joncryl	0.007	**
		W/O modifier vs. with Diisocyanate	0.176	ns
Alfrimal 104 $Al(OH)_3$	Elongation at break [%]	W/O modifier vs. with PhA	0.490	ns
		W/O modifier vs. with Joncryl	0.017	*
		W/O modifier vs. with Diisocyanate	0.022	*
	Tensile strength at break [Mpa]	W/O modifier vs. with PhA	0.353	ns
		W/O modifier vs. with Joncryl	0.035	*
		W/O modifier vs. with Diisocyanate	0.007	**
	Tensile stress at yield [MPa]	W/O modifier vs. with PhA	0.025	*
		W/O modifier vs. with Joncryl	0.653	ns
		W/O modifier vs. with Diisocyanate	0.260	ns
Alfrimal 106 $Al(OH)_3$	Elongation at break [%]	W/O modifier vs. with PhA	0.002	**
		W/O modifier vs. with Joncryl	0.065	ns
		W/O modifier vs. with Diisocyanate	0.080	ns
	Tensile strength at break [Mpa]	W/O modifier vs. with PhA	3.14×10^{-9}	***
		W/O modifier vs. with Joncryl	0.001	***
		W/O modifier vs. with Diisocyanate	5.39×10^{-9}	***
	Tensile stress at yield [MPa]	W/O modifier vs. with PhA	0.005	**
		W/O modifier vs. with Joncryl	0.534	ns
		W/O modifier vs. with Diisocyanate	7.93×10^{-8}	***
Omyacarb 1T-VA $CaCO_3$	Elongation at break [%]	W/O modifier vs. with PhA	0.302	ns
		W/O modifier vs. with Joncryl	0.013	*
		W/O modifier vs. with Diisocyanate	0.167	ns
	Tensile strength at break [Mpa]	W/O modifier vs. with PhA	0.002	**
		W/O modifier vs. with Joncryl	0.685	ns
		W/O modifier vs. with Diisocyanate	0.001	***
	Tensile stress at yield [MPa]	W/O modifier vs. with PhA	0.013	*
		W/O modifier vs. with Joncryl	0.049	*
		W/O modifier vs. with Diisocyanate	2.83×10^{-5}	***
Calprec PR $CaCO_3$	Elongation at break [%]	W/O modifier vs. with PhA	0.351	ns
		W/O modifier vs. with Joncryl	0.476	ns
		W/O modifier vs. with Diisocyanate	0.020	*
	Tensile strength at break [Mpa]	W/O modifier vs. with PhA	0.080	ns
		W/O modifier vs. with Joncryl	0.848	ns

		W/O modifier vs. with Diisocyanate	6.10×10^{-5}	***
	Tensile stress at yield [MPa]	W/O modifier vs. with PhA	0.001	***
		W/O modifier vs. with Joncryl	0.479	ns
		W/O modifier vs. with Diisocyanate	0.045	*
HTP3 Talc	Elongation at break [%]	W/O modifier vs. with PhA	0.014	*
		W/O modifier vs. with Joncryl	2.23×10^{-4}	***
		W/O modifier vs. with Diisocyanate	0.023	*
	Tensile strength at break [Mpa]	W/O modifier vs. with PhA	1.43×10^{-4}	***
		W/O modifier vs. with Joncryl	0.001	***
		W/O modifier vs. with Diisocyanate	2.96×10^{-4}	***
	Tensile stress at yield [MPa]	W/O modifier vs. with PhA	1.54×10^{-4}	***
		W/O modifier vs. with Joncryl	1.65×10^{-4}	***
		W/O modifier vs. with Diisocyanate	0.500	ns
HTP4 Talc	Elongation at break [%]	W/O modifier vs. with PhA	3.75×10^{-4}	***
		W/O modifier vs. with Joncryl	0.468	ns
		W/O modifier vs. with Diisocyanate	0.238	ns
	Tensile strength at break [Mpa]	W/O modifier vs. with PhA	1.27×10^{-4}	***
		W/O modifier vs. with Joncryl	0.038	*
		W/O modifier vs. with Diisocyanate	0.125	ns
	Tensile stress at yield [MPa]	W/O modifier vs. with PhA	1.37×10^{-8}	***
		W/O modifier vs. with Joncryl	0.001	***
		W/O modifier vs. with Diisocyanate	6.02×10^{-7}	***
HTP05c Talc	Elongation at break [%]	W/O modifier vs. with PhA	1.81×10^{-4}	***
		W/O modifier vs. with Joncryl	0.606	ns
		W/O modifier vs. with Diisocyanate	0.058	ns
	Tensile strength at break [Mpa]	W/O modifier vs. with PhA	6.30×10^{-10}	***
		W/O modifier vs. with Joncryl	1.56×10^{-4}	***
		W/O modifier vs. with Diisocyanate	6.25×10^{-8}	***
	Tensile stress at yield [MPa]	W/O modifier vs. with PhA	9.31×10^{-7}	***
		W/O modifier vs. with Joncryl	2.36×10^{-5}	***
		W/O modifier vs. with Diisocyanate	4.99×10^{-7}	***
Ultrasil 7000GR SiO_2	Elongation at break [%]	W/O modifier vs. with PhA	0.361	ns
		W/O modifier vs. with Joncryl	0.026	*
		W/O modifier vs. with Diisocyanate	0.025	*
	Tensile strength at break [Mpa]	W/O modifier vs. with PhA	0.739	ns
		W/O modifier vs. with Joncryl	0.844	ns
		W/O modifier vs. with Diisocyanate	0.048	*
	Tensile stress at yield [MPa]	W/O modifier vs. with PhA	0.269	ns
		W/O modifier vs. with Joncryl	-	-
		W/O modifier vs. with Diisocyanate	0.040	*

Note: ns – not significant; * – $p < 0.05$ (significant); ** – $p < 0.01$ (highly significant); *** – $p < 0.001$ (very highly significant).

FTIR analysis of filler–reactive modifier combinations

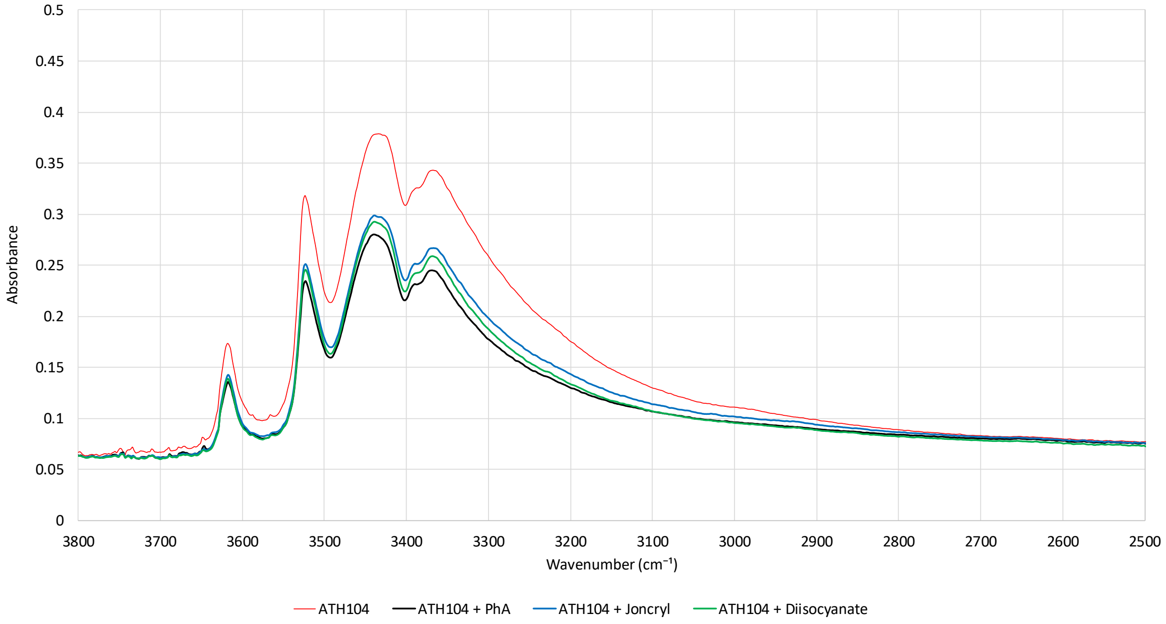

Figure S19. FTIR spectra of Alfrimal 104 ($Al(OH)_3$) powder and its mixtures with PhA, Joncryl, and diisocyanate after thermal treatment at 180 °C for 5 min.

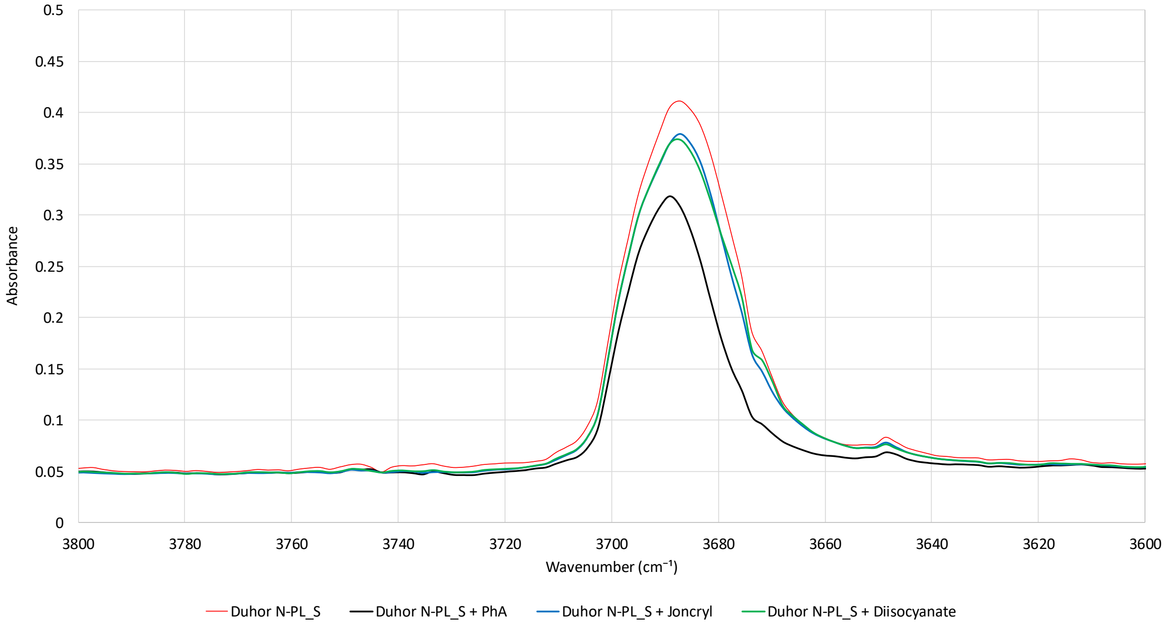

Figure S20. FTIR spectra of Duhor N-PL/S ($Mg(OH)_2$) powder and its mixtures with PhA, Joncryl, and diisocyanate after thermal treatment at 180 °C for 5 min.
